# A 5-D Implementation of FGM for the Large Eddy Simulation of a Stratified Swirled Flame with Heat Loss in a Gas Turbine Combustor

**DOI:** 10.1007/s10494-016-9777-7

**Published:** 2016-11-04

**Authors:** A. Donini, R. J. M. Bastiaans, J. A. van Oijen, L. P. H. de Goey

**Affiliations:** Combustion Technology Group, Department of Mechanical Engineering, Eindhoven University of Technology, Den Dolech 2, 5612 AZ Eindhoven, Netherlands

**Keywords:** FGM, Swirl, Gas turbines, Heat loss, Stratification, LES

## Abstract

Numerical simulations are foreseen to provide a tremendous increase in gas-turbine burners efficiency in the near future. Modern developments in numerical schemes, turbulence models and the consistent increase of computing power allow Large Eddy Simulation (LES) to be applied to real cold flow industrial applications. However, the detailed simulation of the gas-turbine combustion process remains still prohibited because of its enormous computational cost. Several numerical models have been developed in order to reduce the costs of flame simulations for engineering applications. In this paper, the Flamelet-Generated Manifold (FGM) chemistry reduction technique is implemented and progressively extended for the inclusion of all the combustion features that are typically observed in stationary gas-turbine combustion. These consist of stratification effects, heat loss and turbulence. Three control variables are included for the chemistry representation: the reaction evolution is described by the reaction progress variable, the heat loss is described by the enthalpy and the stratification effect is expressed by the mixture fraction. The interaction between chemistry and turbulence is considered through a presumed beta-shaped probability density function (PDF) approach, which is considered for progress variable and mixture fraction, finally attaining a 5-D manifold. The application of FGM in combination with heat loss, fuel stratification and turbulence has never been studied in literature. To this aim, a highly turbulent and swirling flame in a gas turbine combustor is computed by means of the present 5-D FGM implementation coupled to an LES turbulence model, and the results are compared with experimental data. In general, the model gives a rather good agreement with experimental data. It is shown that the inclusion of heat loss strongly enhances the temperature predictions in the whole burner and leads to greatly improved NO predictions. The use of FGM as a combustion model shows that combustion features at gas turbine conditions can be satisfactorily reproduced with a reasonable computational effort. The implemented combustion model retains most of the physical accuracy of a detailed simulation while drastically reducing its computational time, paving the way for new developments of alternative fuel usage in a cleaner and more efficient combustion.

## Introduction

A predominant part of the energy needed by the world is obtained by the combustion of fossil fuels. Although alternative sources are developing at a fast rate, energy production by means of combustion is expected to remain prevailing in the next decades [1]. This forecast especially applies to high power density applications, such as gas turbines. Gas turbines, especially in combined cycle applications, are one of the most important and widely-used energy power generation technologies in the world today. This is because using gas turbines, large scale, high efficiency, low cost and low emission energy production is possible. In the design of a gas turbine, the combustion system plays a crucial role in determining several engine operating characteristics. The main technological drivers during the design of a modern gas turbine combustion system are: efficiency, pollutant emissions, stability and ability to burn different fuels. Accomplishing an advantageous balance of these key-points is a great engineering challenge. Conventional combustion technical know-how is not sufficient in order to achieve these concurring demands. However, the development of techniques such as lean premixed combustion have the potential to meet these strict requirements [2]. In industry the development of clean and efficient technologies for the combustion process is achieved by a combination of experimental and numerical research. Experiments are extremely expensive and time consuming as well, whereas modern engineering trends tend towards shorter and more efficient design cycles. A great reduction of the costs could be made by maximizing the usage of simulations in the design phase. These reasons, together with the persisting advance in the computer technology, are sufficient to elucidate the phenomenal growth of interest in Computational Fluid Dynamics (CFD) of reacting flows in the last few decades. Modern developments in numerical schemes, turbulence models and the consistent increase of computing power allow even Large Eddy Simulation (LES) to be applied to real cold flow industrial applications. In fact, Reynolds Averaged Navier-Stokes (RANS) simulations are currently a primary tool for the gas turbine burner design, but over the last few years LES has undergone considerable development and it is starting to make an important contribution to the design process. However, the detailed simulation of the combustion process remains still prohibited because of the enormous computational cost of the combustion description, and its modeling yet represents a challenging task. In fact, the interaction of turbulence, chemical reactions and thermodynamics in reacting flows is of exceptional complexity.

The objective of this paper is the description of the development, implementation and validation of a methodology for the simulation of turbulent reacting flows at a reasonable CPU cost, with a specific application to gas-turbine combustion. To this end the Flamelet-Generated Manifold chemistry reduction technique [3, 4] is implemented in the LES context and adopted for the simulation of a gas turbine combustion system. FGM is a method which provides the means to reduce the computational costs of chemistry models by several orders of magnitude without sacrificing too much accuracy. Hereby FGM enables the application of reliable chemistry mechanisms in CFD simulations of combustion processes. With the purpose of including many aspects in the numerical modeling of gas turbine combustion systems (this automatically includes stretch to first order approximation, [5] and [6]), in this paper, the FGM model is implemented and applied to turbulent cooled stratified premixed flames by means of a 5-D manifold. A large part of premixed combustion models assume that fuel and oxidizer are fully premixed prior to the combustion process. However, this is often not the case for many engineering applications (such as gas turbines) in which fuel and oxidizer may not be perfectly mixed prior to combustion. This type of flames are defined as partially premixed, which can be considered as an intermediate combustion type between premixed and diffusion. Furthermore, if the flame mode is premixed (diffusion flame does not occur because of a limited gradient in mixture fraction) the reaction can be referred to as stratified premixed combustion. In order to include this effect and its combination with heat loss, three control variables are included for the chemistry representation: the reaction evolution is described by the reaction progress variable, the heat loss is described by enthalpy and the stratification effect is expressed by the mixture fraction. The interaction between chemistry and turbulence is considered through a presumed probability density function (PDF) approach, which is considered for progress variable and mixture fraction. This results in two extra control variables: progress variable variance and mixture fraction variance. The resulting turbulent manifold is five-dimensional, in which the dimensions are progress variable, enthalpy, mixture fraction, progress variable variance and mixture fraction variance.

In previous studies, a number of LES flow prediction studies on swirled configurations are performed with the aim to provide an evaluation of LES codes on flows typical of real burners, e.g. [7–16]. A series of LES studies have been published on the subject of non-adiabatic stratified flames stabilization [17–24], also investigating the impact of the turbulent combustion closure on a non-adiabatic stratified flame [25]. There are few of studies that employ high-dimensional manifolds. Important and quite early ones are [26, 27], applying five dimensions as well. They used a progress variable, the mixture fraction and three (co-)variances or (cross-)dissipation rates. However, the current application of FGM for LES in complex geometry with heat loss, mixture stratification and elevated Reynolds number is still unexplored. For validation purposes, in this paper a highly turbulent and swirling flame in a gas turbine model combustor is computed by means of the above described 5-D FGM implementation. This gas turbine model combustor (a modified version of an original aero gas turbine burner) as extensively studied experimentally e.g. in [28, 29], represents a fully suitable test case for verification and validation of the model, given the challenging complexity of the flow and the comprehensive availability of experimental measurements set.

## Combustion Modeling: the Flamelet-Generated Manifold Method

The detailed simulation, resolving every scale of aerodynamic motion and chemistry (DNS with detailed chemistry) of an entire practical combustion equipment is prohibited because of the current and future limitations in computing power. The support of massive parallel computing facilities has recently enabled the first attempts of Direct Numerical Simulation (DNS) of real life flames [30], endorsed in specific cases by local mesh refinement methods [31, 32]. The use of detailed kinetics and the connected number of 53 species, would make it computationally very expensive. Nonetheless, the DNS of a reacting flow can only be done for limited and isolated turbulent premixed combustion problems. Combustion models aim to reduce the aforementioned computational requirements of flame simulations mainly by a reduction of the number of equations which need to be solved for the reaction, without significantly compromising the quality of results. In the last decades two main routes^1^ have been followed in combustion science to model the detailed dynamics and structure of chemically reacting flows: chemical reduction techniques, and laminar flamelet models.

Chemical reduction techniques are based on the idea that chemicals processes are mainly determined by a small number of slow reactions, and aim to reduce the computational requirements by simplifying the chemical reaction model accordingly. Steady state species are therefore removed to reduce the number of transport equations and to decouple the smallest chemical scales. Typical example of this technique is the reduced chemical reaction mechanisms DRM19 [33]. This type of reduced mechanisms are developed for a specific range of conditions (including extinction and/or auto-ignition behaviour), and their applicability is therefore limited to these cases. It must be remarked that they might include (a better) extinctio and/or auto-ignition behaviour. Outside the specific range of applicability the relevant combustion chemistry is no more included, and unrealistic results may be obtained. Indeed LES contributes as well to the reduction of the computational cost of the simulations presented in this paper. By applying LES, computational reduction can be estimated of a factor 10 or more in each direction. However, the magnitude of computational effort reduction that the chemistry model provides is also very important, given the large reduction of equations which need to be solved during runtime for the chemistry alone (down from 53 to 3 in the case of the current paper).

This is demonstrated in the works cited in Section 2, in which studies were conducted in order to quantify the computational advantage of FGM in the framework of DNS. This issue, on which is the largest effect, could be subject of a further scientific discussion. However, most industrial flame simulations require a detailed description of the reaction kinetics (e.g. for the pollutant emission prediction) and therefore simplified reaction models might not always be adequate. The observation that a small number of rate-limiting reactions dictate the overall reaction progress allow the introduction of the low dimensional manifold concept. Such a manifold is assumed to exist, and it is assumed to be parameterized by a small number of slow evolving variables. For these slow evolving variables a transport equation has to be solved, while the other variables can be retrieved from the manifold. The best known mathematically based reduction methods of this category are 1) the one proposed in [34], in which the slow and fast variables are determined by the user, 2) the Intrinsic Low-Dimensional Manifold (ILDM) method [35] in which the quasi steady state variables are determined by an analysis of the eigen-structure of the Jacobian of the local chemical source terms, and 3) the Computational Single Perturbation (CSP) technique [36]. In these methods diffusion is fully neglected, hence only the chemical source term combined with advection are taken into account. This represents a limit when diffusive processes become important. This occurs in locally cooled regions or in extinction and re-ignition zones, in which the dimensionality of the chemical manifold rapidly increases and incorrect mass fractions are predicted [3]. Furthermore the time-scales associated with the formation of slow evolving species (e.g. CO) can be associated with convective-diffusive processes [35], resulting in a poor prediction of such species.

Laminar flamelet models introduce a different approach to the flame description. In these methods it is assumed that the turbulent flame brush consists of an ensemble of discrete, steady laminar one-dimensional flames [37], referred to as *flamelets*. This assumption means that thin quasi one-dimensional flame structures can be isolated in multi-dimensional flames, and consequently a main direction for the gradients of the thermochemical variables can be identified. An asymptotic analysis leads to the derivation of flamelet equations, which describe the inner structure of the laminar flame, thus decoupling the chemistry from the flow field. The multi-dimensional flame can therefore be seen as a continuous ensemble of flamelets embedded in the flow. A scale analysis allows to extend this assumption for turbulent flames as well [38]. In fact, quasi one-dimensional flame structures exist when turbulent eddies are not able to break down the inner reaction layer, i.e. in the corrugated flamelet regime and in the thin reaction zones regime [39]. In these regimes the location of the flame can be detected by iso-surfaces of a certain scalars, and in this location the flamelet (i.e. the solution of the flamelet equations) is reattached to the turbulent flow field.

Flamelet-Generated Manifold (FGM) [3] and Flamelet Prolongation of ILDM (FPI) [40] are two tabulated approaches developed independently, but conceptually very similar. These methods introduce the identification of a low-dimensional manifold based on flamelet structures and can therefore be considered as a hybrid of the classic flamelet models and chemistry reduction techniques discussed above [39].

In FGM a database of thermochemical variables is generated in a pre-processing stage for given initial conditions, and stored as a function of a small number of controlling variables. During the flame simulation only the transport equations for the control variables need to be solved, and all the dependent thermochemical variables can be retrieved from the pre-calculated chemistry manifold. In the case that detailed solutions of one-dimensional laminar premixed flames are used as the basis for the tabulation, FGM and FPI are essentially identical, and the remaining differences are merely technical. The chemistry manifold is stored as a common practice in a tabulated form, although several improved storage methods like Artificial Neural Networks [41, 42], high-order orthogonal polynomial parametrization [43] or in situ adaptive tabulation [44] have been proposed in the past years. The FGM technique has the admirable advantage of considerably reducing the computational cost of combustion simulations [3, 45], while keeping the accuracy to high levels. A key advantage of FGM is in fact the capability to predict minor species in a consistent way [4]. The FGM technique has proven to be very accurate for laminar premixed Bunsen flames including heat loss presence [3, 45, 46], preferential diffusive effects [47], highly stretched premixed counter-flow flames [5] and confined triple flames [6]. This technique performed well also in DNS of a turbulent expanding flame [48], showing that a single control variable can give accurate predictions on the local mass burning rate. Furthermore, the approach has proven to be appropriate also for the computation of turbulent flames with heat loss [49] as well as for turbulent partially premixed flames [50, 51]. However, this technique has never been extended in order to include these last effects in combination (stratification, heat loss and turbulence). At the best of the authors’ knowledge, the application of FGM in combination with heat loss, fuel stratification and turbulence has never been studied in literature. Furthermore, the application of FGM in the LES context for the simulation of a gas turbine burner is still not fully explored and fascinating from a scientific and engineering point of view.

### The FGM database generation

In this study cooled stratified premixed methane/air turbulent combustion is considered, at atmospheric pressure conditions. These effects are represented by a 5-D FGM in which the control variables are represented by the progress variable $\widetilde {\mathcal {Y}}$, enthalpy $\widetilde {h}$, mixture fraction $\widetilde {Z}$, progress variable variance $\widetilde {\mathcal {Y}^{\prime \prime 2}}$ and mixture fraction variance $\widetilde {Z^{\prime \prime 2}}$. The database creation is described in the present subsection.

The progress variable $\mathcal {Y}$ quantitatively defines the transition from fresh mixture to burned gases. The progress variable is defined as a linear combination of species mass fraction:

1 $$ \mathcal{Y} = \sum\limits_{i=1}^{N_{s}} \alpha_{i} Y_{i} ,  $$

where *Y*_*i*_ is the mass fraction of specie *i* and *N*_*s*_ the total number of species. The weighting coefficients *α*_*i*_ are arbitrarily chosen, with the only restriction of ensuring a monotonic profile of $\mathcal {Y}$ in the whole interval between the unburned mixture and the chemical equilibrium. For the calculations described in this paper it is chosen

- $\alpha _{CO_{2}} = M_{CO_{2}}^{-1}$,
- $\alpha _{H_{2}} = M_{H_{2}}^{-1}$,
- $\alpha _{H_{2}O} = M_{H_{2}O}^{-1}$,
- $\alpha _{O_{2}} = -M_{O_{2}}^{-1}$,
- $\alpha _{i} = 0 \ \forall \ i \not \in \left \{ CO_{2}, H_{2}, H_{2}O, O_{2} \right \}$,

in which *M*_*i*_ is the molecular mass of species *i*. This choice is made in order to optimize the chemistry resolution, although a number of methods for the automated optimization of the progress variable definition have been recently presented, e.g. [52]. For practical convenience the progress variable is scaled (normalized) between 0 (fresh mixture) and 1 (chemical equilibrium) [53]. The normalized progress variable is adopted here for clarity in the visual representation of the results.

In the case under study in this paper the enthalpy is not conserved throughout the domain because of the heat loss to the combustion chamber walls, and by means of radiative effects. Furthermore, fuel and oxidizer are not perfectly mixed prior to combustion, yet conserving the premixed flame mode. In order to take these two effects into account in the tabulation process, a set of laminar flamelets have to be solved for different values of enthalpy *h* and mixture fraction *Z*, introducing these two as control variables. Full kinetics flamelet solutions are obtained by means of a specialized 1D flame code (Chem1D [54]), coupled with the GRI-Mech 3.0 mechanism [55], which consists of 325 elementary reactions between 53 species with hydrocarbons up to propane. Unity Lewis number is imposed along the flamelets, neglecting differential diffusive effects. The underlying reason for this approximation is that at high turbulence levels diffusion of species and temperature is dominated by turbulent mixing, resulting in an effective unity Lewis number [56, 57], and allowing a much simpler formulation of the FGM equations [53]. At first, the flamelets are computed as steady, fully premixed, flat flames (stretch-less: all perturbations from 1D flat-flame behavior are neglected), for a given pressure, composition and temperature of the inlet mixture. At the burned side (chemical equilibrium) Neumann type boundary conditions are imposed instead.

The enthalpy is taken into account in the flamelet database creation process by the generation of an enthalpy-decreasing set of flamelets. This procedure might be performed in a number of different ways, but the most straightforward are:

- decreasing the enthalpy of free adiabatic flamelets by simply diminishing the inlet temperature, therefore a series of flamelets is computed for different values of $h_{-\infty }$,
- calculation of burner-stabilized flamelets, and therefore imposing a certain increasing amount of heat loss to the burner.

**Fig. 1:**
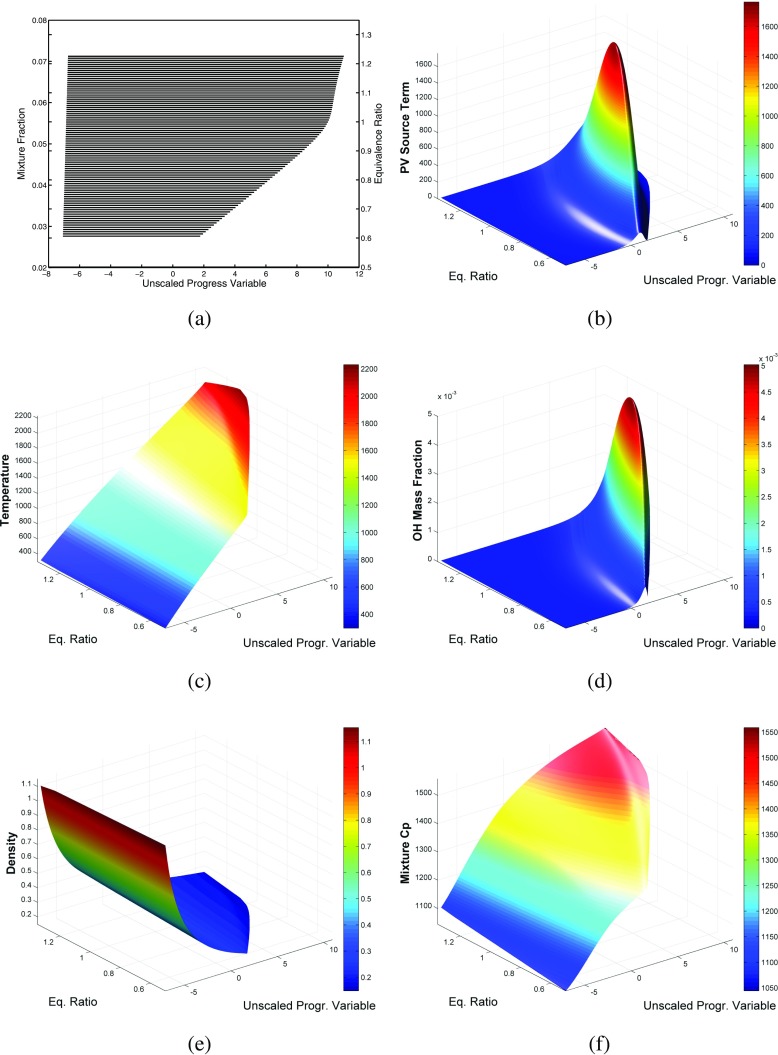
Representations of the laminar manifold at the highest enthalpy level. **a** Mixture fraction along the flamelets composing the manifold. **b** Progress variable source term [kg m ^−3^ s ^−1^] profile along the manifold. **c** Temperature [K]. **d** OH mass fraction. **e** Density [kg m ^−3^]. **f** Specific heat capacity at constant pressure of the mixture [J kg ^−1^ K ^−1^]

Here both methods are used in order to have a fairly complete manifold, in fact burner-stabilized flamelets allow to impose very high values of cooling. The first method is used for low values of cooling until unrealistic values of inlet temperature are reached (limits in the reaction mechanism validity [55]), then the second method is adopted. It has been proven that the choice of the enthalpy-decrease method for the tabulation procedure has negligible influence on the final result [5]. Note that this method for the inclusion of enthalpy in the creation of the database makes use of constant enthalpy flamelets. Therefore, this approach might not be valid (or might as well, since this topic has never been addressed in literature) in the case that the cooling takes place in the flame active region. However, this consideration is not relevant to the flame under examination of this study, since the flame active region is sufficiently far from the walls (which are the main contributors for the heat loss). In the so called sub-cooled region (the small area in which the enthalpy is lower than the coldest flamelet) an extrapolation is performed as described in [53]. Even if such a procedure represents an approximation, it performs quite well [3]. Favorably, in most practical cases the cooling takes place in the burned gases, which corresponds to manifold entries very close to chemical equilibrium. An overview of the resulting (3-D at this point: $\mathcal {Y}$-*h*-*Z*) laminar manifold can be seen in Fig. 1, where various scalars *ψ* are represented as a function of the progress variable and equivalence ratio for the highest enthalpy value. It has to be noted that here enthalpy is referenced to inlet conditions (*h*=0 at the inlet).

The mixture fraction *Z* is expressed in terms of element mass fractions *Z*_*j*_ of element *j* [58]:

2 $$ Z= \frac{2 M_{H}^{-1} \left( Z_{H}-Z_{H,2}\right)+0.5 M_{C}^{-1} \left( Z_{C}-Z_{C,2}\right) - M_{O}^{-1} \left( Z_{O}-Z_{O,2}\right) }{2 M_{H}^{-1} \left( Z_{H,1}-Z_{H,2}\right)+0.5 M_{C}^{-1} \left( Z_{C,1}-Z_{C,2}\right) - M_{O}^{-1} \left( Z_{O,1}-Z_{O,2}\right) },  $$

in which the subscripts 1 and 2 denote respectively pure fuel and pure oxidizer. *Z* is defined as a linear combination of element mass fractions *Z*_*i*_, and it is therefore conserved throughout the chemical reaction. In partially premixed flames fuel and oxidizer are not perfectly mixed, therefore variations in local element composition occur. In order to take this into account in the tabulation process, the laminar flamelets are solved for different values of mixture fraction. The mixture fraction is varied simply by tuning the mass fraction boundary condition at the inlet (for a representation of the mixture fraction along the flamelets composing the manifold see Fig. 1a). The range of the flamelet solution goes from *ϕ*=0.6 to *ϕ*=1.45, which are close to the flammability limits. Therefore *Z* is introduced as a control variable, where the convenience of this approach is demonstrated in previous studies on partially premixed flames, e.g. [7, 50, 59].

The calculation of the thermodynamic coefficients is performed by means of a mixture averaged approach (for a detailed description see [53]) during the pre-processing stage, and stored in the manifold together with the chemical data.

The turbulence-chemistry interaction is considered for progress variable and mixture fraction by means of a presumed *β*-PDF model [9, 60] Given the rather modest gradients and fluctuations for the enthalpy In the flame zone, a *δ*-function is chosen as a first model for enthalpy. With this model, assuming that the (scaled) progress variable $\mathcal {Y}$ and mixture fraction *Z* are statistically independent in the flame [61], e.g. the source term of the progress variable $\overline { \dot {\omega } }_{\mathcal {Y}}$ is given by

3 $$ \overline{ \dot{\omega} }_{\mathcal{Y}} = \int \int {{ \dot{\omega} }_{\mathcal{Y}} (\mathcal{Y},h,Z) } P(\mathcal{Y},\widetilde{\mathcal{Y}},\widetilde{\mathcal{Y}^{\prime\prime 2}}) P(Z,\widetilde{Z},\widetilde{Z^{\prime\prime 2}}) d\mathcal{Y} dZ, $$

in which the marginal probability distribution *P* is the presumed *β*-PDF. This approach has been successfully applied in a number of previous studies, e.g. [62, 63]. This convolution operation generates an increase of two dimensions in the manifold, which finally reaches the number of five dimensions. The final dimensions for the manifold are: progress variable $\widetilde {\mathcal {Y}}$, enthalpy $\widetilde {h}$, mixture fraction $\widetilde {Z}$, variance of progress variable $\widetilde {\mathcal {Y}^{\prime \prime 2}}$ and variance of mixture fraction $\widetilde {Z^{\prime \prime 2}}$.

**Fig. 2:**
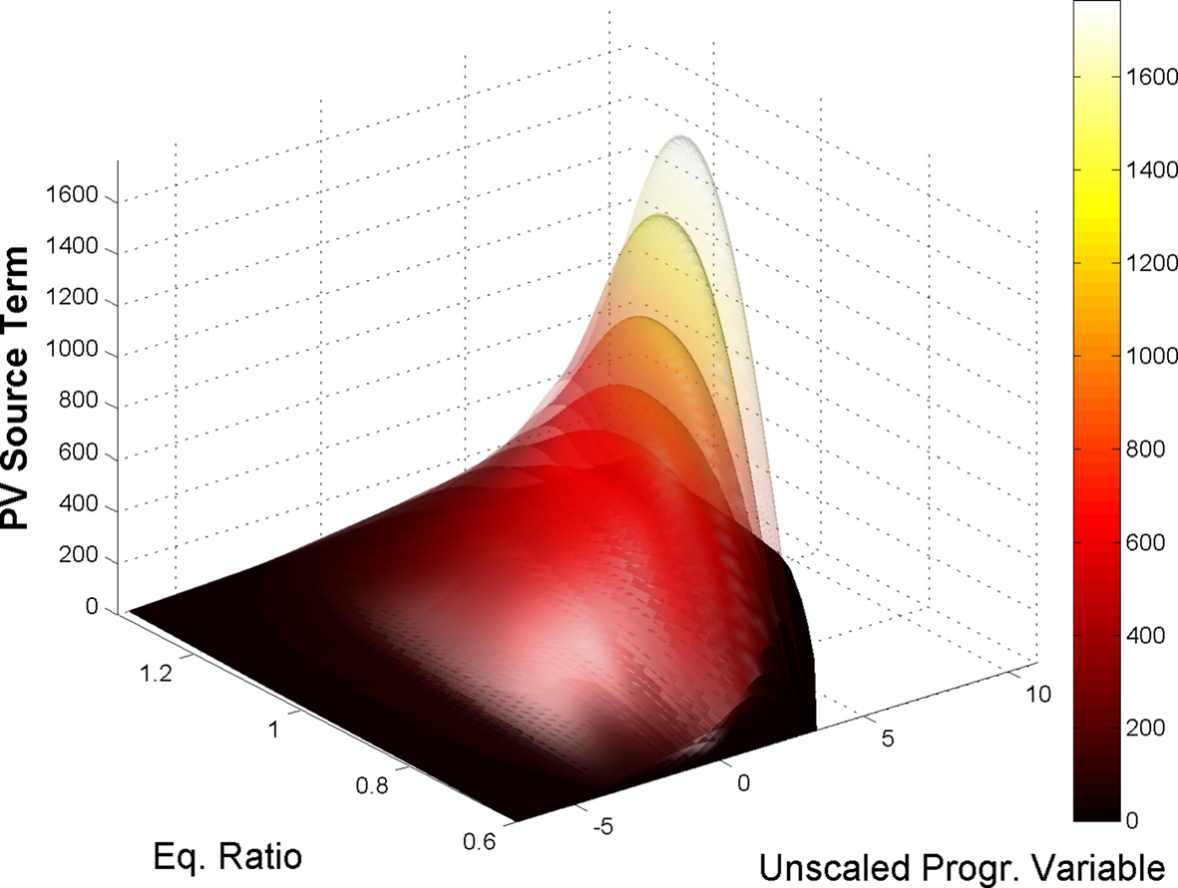
Illustrative representation of the 5D manifold at the highest enthalpy level and zero variance of mixture fraction. The surfaces show the progress variable source term [kg m ^−3^ s ^−1^] profile as a function of unscaled progress variable and equivalence ratio, at different levels of variance of the progress variable, shown with different transparencies

An illustrative overview of the resulting manifold can be seen in Fig. 2, in which the progress variable source term is represented at the maximum level of enthalpy and zero variance of mixture fraction, as a function of the progress variable and equivalence ratio (i.e. mixture fraction, under the assumption of unity Lewis number), for different levels of variance of progress variable (in transparency).

### The storage of the manifold

The tabulation procedure consists in storing the desired thermochemical variables *ψ* (i.e. $\overline {\psi }$ and $\widetilde {\psi }$ as well) in a tabulated form, on a predefined five-dimensional grid for which the $\widetilde {\mathcal {Y}}$-$\widetilde {h}$-$\widetilde {Z}$-$\widetilde {\mathcal {Y}^{\prime \prime 2}}$-$\widetilde {Z^{\prime \prime 2}}$ range is discretized. The number of points adopted for the manifold is $n_{\widetilde {\mathcal {Y}}}=120$, $n_{\widetilde {h}}=30$, $n_{\widetilde {Z}}=40$, $n_{\widetilde {\mathcal {Y}^{\prime \prime 2}}}=10$ and $n_{\widetilde {Z^{\prime \prime 2}}}=10$. The data is stored by means of an equidistantly spaced grid in the $\widetilde {\mathcal {Y}}$, $\widetilde {h}$ and $\widetilde {Z}$ direction. For the two variances $\widetilde {\mathcal {Y}^{\prime \prime 2}}$ and $\widetilde {Z^{\prime \prime 2}}$ a quadratic spacing is adopted in order to have a finer grid close to zero, because of the typically steeper gradients of this region.

### The solution stage

The tabulated FGM database can now be linked to a standard CFD solver. The CFD solver, in addition to the momentum and continuity equations, solves three extra transport equations: the progress variable, enthalpy and mixture fraction transport equation. A standard eddy viscosity model is adopted in order to close the non linear terms of these equations [64]. In this way, the equation for the general transported scalar *ψ* can be written as

4 $$ \frac{\partial \overline{\rho} \widetilde{\psi}}{\partial t} + \frac{\partial}{\partial x_{j}} \left( \overline{\rho} \widetilde{u}_{j} \widetilde{\psi} \right) = \frac{\partial}{\partial x_{j}} \left[ \left( \frac{\lambda}{c_{p}} + \frac{\mu_{t}}{Sc_{t}} \right) \frac{\partial \widetilde{\psi}}{\partial x_{j}} \right] + \overline{ \dot{\omega} }_{\psi},  $$

in which *ρ* is the mixture density, *μ*_*t*_ the dynamic viscosity of the mixture, *λ* the mixture conductivity, *c*_*p*_ the mixture specific heat capacity at constant pressure and *u*_*j*_ the velocity component in direction *x*_*j*_. The turbulent Schmidt number *S* *c*_*t*_, is assumed to have a fixed value of *S* *c*_*t*_=0.7 [65]. This equation is practically equivalent for the three transported control variables $\mathcal {Y}$, *h* and *Z*, with the only main difference that the source term is non-zero only for the progress variable (i.e. the chemical source term) and for enthalpy because of the radiative heat loss (see Section 3.5). Lewis numbers are assumed to be unity for both progress variable (for detailed methane combustion, unity is a good approximation), enthalpy and mixture fraction. For the turbulent transport, with subgrid advection, modeled by diffusion, a unity Lewis number is in our opinion considered as a good choice. This is open to scientific discussion. Subfilter terms regarding *μ*_*t*_, *λ* and *c*_*p*_ are neglected, which is a common practice in LES [50]. All the thermochemical parameters (e.g. $\overline {\rho }$, *c*_*p*_, *λ*, $\overline { \dot {\omega } }_{\mathcal {Y}}$, $\widetilde {T}$) are retrieved from the FGM database as function of the five control variables $\widetilde {\mathcal {Y}}$, $\widetilde {h}$, $\widetilde {Z}$, $\widetilde {\mathcal {Y}^{\prime \prime 2}}$ and $\widetilde {Z^{\prime \prime 2}}$. The data retrieval from the manifold for given values of the control variables is performed by means of a penta-linear interpolation [53] on the tabulated values.

In addition to the three transported control variables $\mathcal {Y}$, *h* and *Z*, a calculation method is needed for the variances $\widetilde {\mathcal {Y}^{\prime \prime 2}}$ and $\widetilde {\mathcal {Z}^{\prime \prime 2}}$. A suitable, and often used, model is the similarity or gradient-based model [66], which (e.g. for the progress variable) reads:

5 $$ \widetilde{\mathcal{Y}^{\prime\prime 2}} \approx \frac{a^{2} {\Delta}^{2}}{12} \left( \frac{\partial \widetilde{\mathcal{Y}} }{\partial x}\right)^{2} = \alpha {\Delta}^{2} \left( \frac{\partial \widetilde{\mathcal{Y}} }{\partial x}\right)^{2}  $$

where Δ is the filter width, which is considered to be equivalent to the grid resolution. The parameter *a* is assumed to be constant. It can be deduced that the value of *a* should lie between 1 and 2, since $\widetilde {\mathcal {Y}} (1- \widetilde {\mathcal {Y}}) \le \frac {1}{4}$. For smooth fields and in case of second order discretization over an equidistant grid, a Taylor expansion yields *a*=1 and therefore *α*=1/12 [66]. However, previous studies showed that this value may underestimate variances [67], and that values ranging from *α*=0.1 to *α*=0.3 give correct results, depending on the application [50, 67]. Both progress variable and mixture fraction adopt this gradient-based model for the variance.

Furthermore, in order to have a better prediction of the mass fractions of CO and NO, which have a slow production/consumption, two extra transport equations (corresponding to Eq. 4) are solved for these species. Their source term is directly retrieved from the FGM data-base, i.e. for the nitric oxide $\overline {\dot {\omega }_{NO}}=\overline {\dot {\omega }_{NO}(\widetilde {\mathcal {Y}}, \widetilde {h}, \widetilde {Z}, \widetilde {\mathcal {Y}^{\prime \prime 2}}, \widetilde {Z^{\prime \prime 2}})}$. In this way, only the production and consumption of these species is given by the manifold, while their overall amount is computed by the complete transport events. This approach has proven to yield improved nitric oxide mass fraction predictions [50].

## Application to a Gas Turbine Model Burner

### Geometry of the GT model combustor

**Fig. 3:**
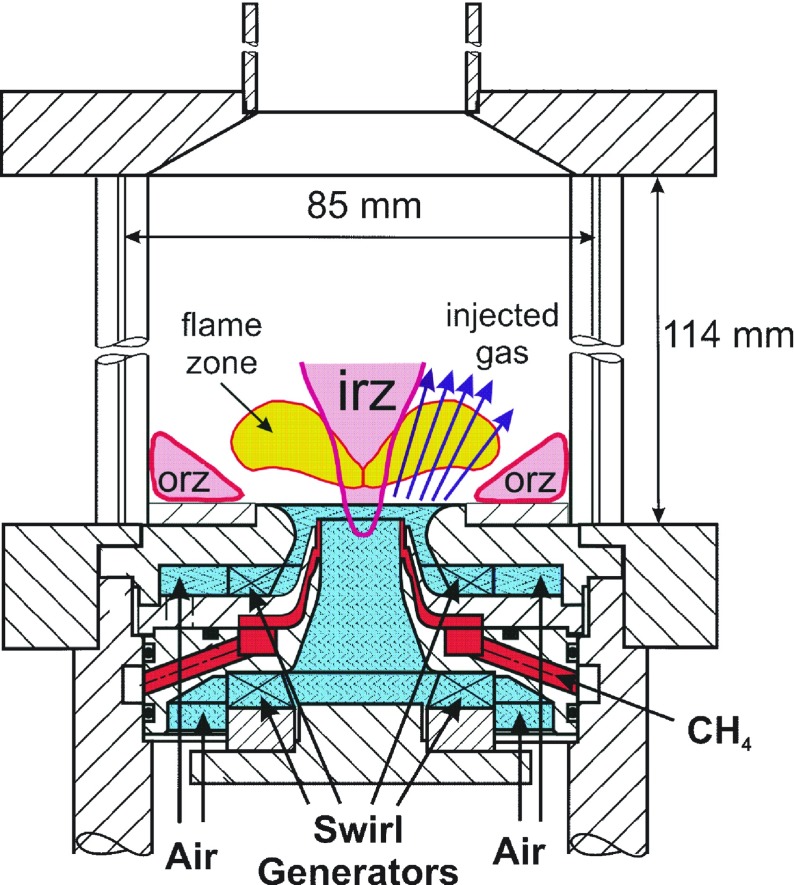
**a** Schematical representation of the gas turbine model combustor geometry, including a drawing of the flow field features and main areas of recirculation: Inner Recirculation Zone (IRZ) and Outer Recirculation Zone (ORZ). From [29, 68]

The highly turbulent and swirling flame in a gas turbine model combustor under study in this work was extensively studied experimentally in [28, 29, 68, 69]. A schematical representation of the burner is shown in Fig. 3 along with the expected flow features. Dry air at room temperature and atmospheric pressure is fed to the combustion chamber through a central nozzle and an annular concentric co-swirling nozzle. The central nozzle has a diameter of 15 mm, while the annular nozzle has an inner diameter of 17 mm and outer diameter of 25 mm, contoured by an outer exit diameter of 40 mm. Non-swirling methane is supplied through a non-swirled annular inlet, radially located between the air inlets. The combustion chamber downstream the nozzle consists of a square section measuring 85 × 85 mm with a height of 114 mm. The plane of exit of fuel and central air nozzle is located 4.5 mm below the outer air nozzle exit plane. The latter is defined as reference height plane $\mathcal {H} = 0$. A conical top plate with a central exhaust tube (diameter 40 mm, length 50 mm) forms the outlet. The burner is a modified version of an aero gas turbine combustor, originally with an air blast nozzle for liquid fuels [70]. In the experiments [28, 29], three different operating conditions are investigated. Among these, the so called *flame A* is selected for the numerical analysis presented here. This operating condition is outlined by the following global parameters: thermal power *P*_*th*_=34.9 kW, air mass flow rate $\dot {m}_{air}=0.01825$ kg s ^−1^, fuel mass flow rate $\dot {m}_{fuel}=0.696$ g s ^−1^, global equivalence ratio *ϕ*_*global*_=0.65 and global adiabatic flame temperature *T*_*ad*,*global*_=1750 K. The split ratio between air mass flowing through the annular and central nozzle is approximately 1.4 [71]. A swirl number *S* may be defined as

6 $$ S=\frac{{{\int}^{R}_{0}} u_{ax} u_{tan} \rho r dr}{R {{\int}^{R}_{0}} u_{ax}^{2} \rho r dr}, $$

in which *u*_*ax*_ is the axial velocity, *u*_*tan*_ the circumferential velocity, *r* the radius and *R* the maximum radius of the nozzle exit. The swirl number calculated from the experimental velocity profile just above the nozzle exit is *S*=0.9. The nozzle Reynolds number calculated on the cold inflow and the minimum outer nozzle diameter (25 mm) is approximately 58 000 [28]. These settings have been reproduced in the simulations, as described in the following section. Detailed heat loss information is not directly available from the experiments, however the presence of non insulated optical accessible burner walls leads to safely assume a certain amount of heat loss. In addition, heat loss presence is confirmed in the experimental results [29]. Heat loss to the walls is imposed in the simulations presented in this paper by enforcing an isothermal temperature *T*_*w*_=800 K to the walls. The specific temperature *T*_*w*_ is a simplified approximation, and it is chosen by analyzing and extrapolating the experimental temperatures close to the walls in the lower part of the combustion chamber. The extrapolated value certainly represents an estimation, yet it is interesting to investigate its effect on the flame. In the experiments of [28, 29] the flow field is measured by Laser Doppler Velocimetry (LDV), the flame structures are visualized by planar laser-induced fluorescence (PLIF) of OH and CH radicals, and the major species concentrations, temperature, and mixture fraction are determined by laser Raman scattering. The uncertainty of the velocity measurements obtained by this technique is estimated to be ±0.3 m/s, while the overall accuracy of the measurement of species concentrations and temperatures with this method is estimated to be approximately within 4 %, measured during the calibration procedure [29].

### Numerical discretization

The three dimensional computational domain consists of a simplified (however fully consistent) representation of the experimental burner. The plenum and air swirlers are not included in the computational domain, in order to keep the computational costs to a reasonable level. The inlet boundary conditions are set at the outlet of the swirlers, with flow angle and mass flow rate consistent with the experimental setup. The mesh is composed of 9 400 000 hexahedral/tetrahedral elements over 6 300 000 nodes, featuring an adequate refinement on the inlet ducts and flame zone. Furthermore, five prismatic layers are imposed at the wall in order to accommodate the preferential orientation of the flow at the boundary layer. This feature is essential in order to capture a correct wall friction and heat flux at the wall. The aspect ratio of the elements in the prism layers is consistently minor than 8, which is in agreement with the remarks of [72]. In addition, for the mesh at the inlet and dump plane, the maximum value of *y* ^+^ is always below 5. The ratio of the turbulent sub-grid viscosity to the molecular viscosity has maximum values of approximately 3.5 in the flame region, whereas maximum values of 20 occur at the vortex core inside the inlet. This ratio is not ideal, however it can be considered acceptable [73]. Furthermore, the ratio of the resolved to the total turbulent kinetic energy is always higher than 75 % [64, 73] of the total energy on the flame zone, which confirms that the grid resolution is adequate.

### Numerical setup

The simulations here presented are performed with the general purpose fluid-dynamics program Ansys-CFX [74], in which the equations are solved by means of a conservative vertex-centered finite-element-based control volume method. The algorithm of the software is implicit, second order accurate in space and time and the solver chosen for the computations of this work is (low-Ma) incompressible. A custom framework for the FGM coupling has been created for the solver. This includes a table upload to the parallel stack memory and an efficient data retrieval with multi-dimensional interpolation, with an automatic management of the boundaries of the tabulated data. For extended details regarding this topic please consult [53]. The approach used for turbulence is LES with Smagorinsky sub-grid scale model [75]. The time-step adopted for the calculations is Δ*t*=2⋅10^−6^ s, which is chosen in order to have a CFL number [76] lower than 0.5 in the entire domain. The total time of the simulation run for the averaging of the flow is *t*_*tot*_=0.09 s. This corresponds to approximately 16 flow-through times, in which the flow-through time is calculated on the combustion chamber and exhaust pipe length.

### Turbulent inlet condition

The generation of proper turbulent inflow conditions is widely studied in literature [77–80]. A particularly successful approach is to consider the inflow conditions as deterministic by extracting the boundary data from complete time series of numerical simulations or experimental measurements [77, 78], or by adding modeled turbulent fluctuations to limited measurements [79]. In general, however, the deterministic approaches are not always practical or feasible with arbitrarily complex flow configurations and therefore have limited applicability in the context of turbulence modeling. A more convenient approach is to generate realistic turbulent inlet conditions synthetically. However, the generation of realistic turbulent fluctuations is puzzling, due to the chaotic and strongly non-linear nature of turbulence. Nevertheless, a class of techniques which is aimed to simulate correlated stochastic flow fields exists. The simplest stochastic technique is to use a random inlet signal with a desired probability distribution [81, 82], however various studies have questioned the use of random inlet boundaries as they do not reproduce the characteristics of turbulent flow [83]. More successful approaches for random flow generation are based on a method of synthesizing divergence-free vector fields, e.g. from a Fourier harmonics sample [80] or by a random two-dimensional vortex method [84]. Although the random approach is effective and computationally convenient, this technique is limited in prescribing user defined correlation and higher order statistics. A more robust class of techniques is known as the WAWS spectral representation method [85]. This approach is a stochastic technique capable of simulating multivariate, multidimensional, non-homogeneous stochastic fields with evolutionary power spectra and arbitrary probability functions.

In the present study the turbulence at the inlet boundary conditions is generated by means of the WAWS stochastic technique, in which the fluctuating signal is reconstructed from its Fourier components that are extracted from a modeled energy density spectrum [86]. The inflow velocity in the WAWS method is calculated as:

7 $$ u_{j}(t)=u_{\infty}+\sum\limits_{m=1}^{M} \sqrt{2} u_{m} sin(\omega_{m} t + \varphi_{m}), $$

in which the *M* frequencies are defined as *ω*_*m*_ = *m*Δ_*ω*_, Δ_*ω*_ = *ω*_*u*_/*M* is the frequency spacing, *ω*_*u*_ is the upper cutoff frequency, and the total number of frequencies *M* in the spectrum is 10. The upper cutoff frequency is calculated here as the frequency of an eddy of dimension 5 *η*, in which the Kolmogorov scale *η* determined by assuming homogeneous isotropic turbulence [64]. The velocity *u*_*m*_ is the frequency specific average velocity of the sinus wave and is calculated from the Fourier components. These are obtained assuming a homogeneous isotropic turbulent power spectrum for the inlet fluctuations. The Fourier components Ω at each frequency are calculated as

8 $$ {\Omega}=\sum\limits_{m=1}^{M} \omega_{m}^{-\frac{5}{3}}. $$

Having the total kinetic energy $E \propto {\Omega }$, the frequency specific average velocity *u*_*m*_ can be calculated as

9 $$ u_{m}=\sqrt{\frac{\omega_{m}^{-\frac{5}{3}}}{\Omega} E}. $$

The parameter *φ*_*m*_ is an independent random phase offset uniformly distributed between 0 and 2 *π*, in order to ensure frequency waves are out of phase. The inlet turbulent kinetic energy *E* is calculated in a *k*−*𝜖* fashion [64]:

10 $$ E=\frac{3}{2} \left( \overline{u} I \right)^{2}, $$

in which the turbulence intensity *I* is defined as the ratio of the root-mean-square of the velocity fluctuations $u^{\prime }$ to the mean free stream velocity $\overline {u}$. The value of the turbulent intensity chosen for the present study is 1 % for the fuel inlet and 5 % for the two air inlets. The injected turbulence level has been chosen after testing on cold flow simulations, and by comparison with experimental data.

### Radiative heat loss model

In the gas turbine model burner considered in this paper it can be expected that the gaseous thermal radiation reduces the local temperature peaks significantly. In general, the inclusion of this effect on the combustion model may give a more realistic temperature prediction, which is important especially for achieving a correct prediction of temperature-sensitive pollutants such as NO. Hence, it is chosen to include a radiation model within the present FGM approach.

**Fig. 4:**
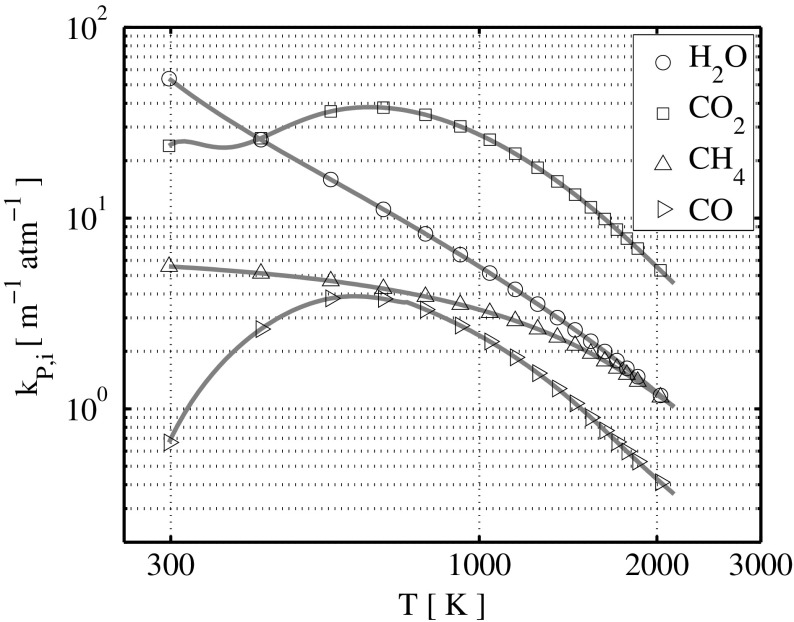
Representation of the Plank mean absorption coefficients *k*_*P*,*i*_ for a methane-air premixed flame with equivalence ratio *ϕ*=0.9. Each line represents a different species, as depicted in the legend. Marker positions are not representative of data points, they are included only for a labeling purpose

**Fig. 5:**
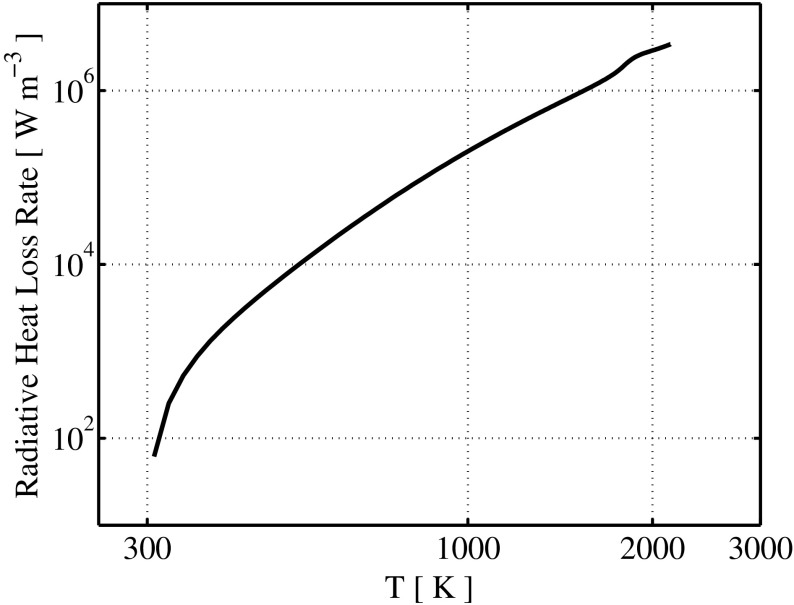
Radiative heat loss volumetric rate *E*_*q*_ calculated by means of Eq. 11, for a methane-air premixed flame with equivalence ratio *ϕ*=0.9

A number of radiation modeling approaches are reported in literature [87] to account for heat loss within numerical investigations. Several different approaches are available also for radiation modeling within the flamelet concept, e.g. [88–90]. The extra feature desired here is to have a radiative model which exploits the advantage of having a tabulated FGM database. Furthermore, a simplified model is desired in order to include the effect of radiation in turbulent combustion with the FGM model without significantly increasing the computational cost. It is therefore chosen to model radiative enthalpy loss as described in [91, 92], using the assumption of optically thin radiative transfer between a given fluid element in the flame and the cold surroundings. The optical-thin limit corresponds to a situation in which the radiating waves have a small path-length through the mixture, i.e. a relatively small gas volume and burner size, atmospheric pressure and each radiating point source has an unobstructed isotropic prospect of the cold surroundings, hence no radiation absorption is considered. This assumption should yield a good approximation of the true radiative effects in methane/air premixed flames [92]. Under the optically thin assumption, the radiative emission per unit volume may be calculated as:

11 $$ E_{q}(T) = 4 \sigma \sum\limits_{i}^{N_{s}} p_{i} k_{P,i} \left( T^{4} - {T_{b}^{4}} \right),  $$

where *σ* is the Stefan-Boltzmann constant, *p*_*i*_ is the partial pressure of species *i*, *N*_*s*_ the number of species, *k*_*P*,*i*_ the Plank mean absorption coefficient of species *i*, *T* the local temperature of the mixture and *T*_*b*_ the background environmental temperature. Here the background temperature is included in order to avoid an unphysical cooling of the mixture below the temperature of the surroundings. Not all the species have a relevant contribution to the radiative emission process, and in practice the most important species for methane combustion are CO _2_, H _2_O, CH _4_ and CO [87], in which CH _4_ and CO are responsible only for a smaller contribution. Only these four species are therefore chosen for the calculations described here. Their Plank mean absorption coefficients *k*_*P*,*i*_ are calculated in a polynomial form as function of temperature [91], for which the coefficients are retrieved in a tabulated form [92]. The resulting coefficients *k*_*P*,*i*_ are shown in Fig. 4 for a laminar methane-air premixed flame with equivalence ratio of *ϕ*=0.9. The corresponding radiative emission loss calculated by means of Eq. 11 is shown in Fig. 5.

The radiative emission *E*_*q*_ is calculated during the pre-processing stage, and conveniently stored in the FGM together with the chemical and transport data. The turbulence-interaction is taken into account by means of the same presumed *β*-PDF model described in Section 2.1. The coefficient is added to the transport equation of enthalpy, in the form of a negative source term, which is directly retrieved from the FGM data-base, e.g.

12 $$ \overline{E_{q}}=\overline{E_{q}(\widetilde{\mathcal{Y}}, \widetilde{h}, \widetilde{Z}, \widetilde{\mathcal{Y}^{\prime\prime 2}}, \widetilde{Z^{\prime\prime 2}})}. $$

This modeling approach is very practical, since it does not affect the computational cost (only marginally during the pre-processing stage).

## Results

### Flame and flow features

**Fig. 6:**
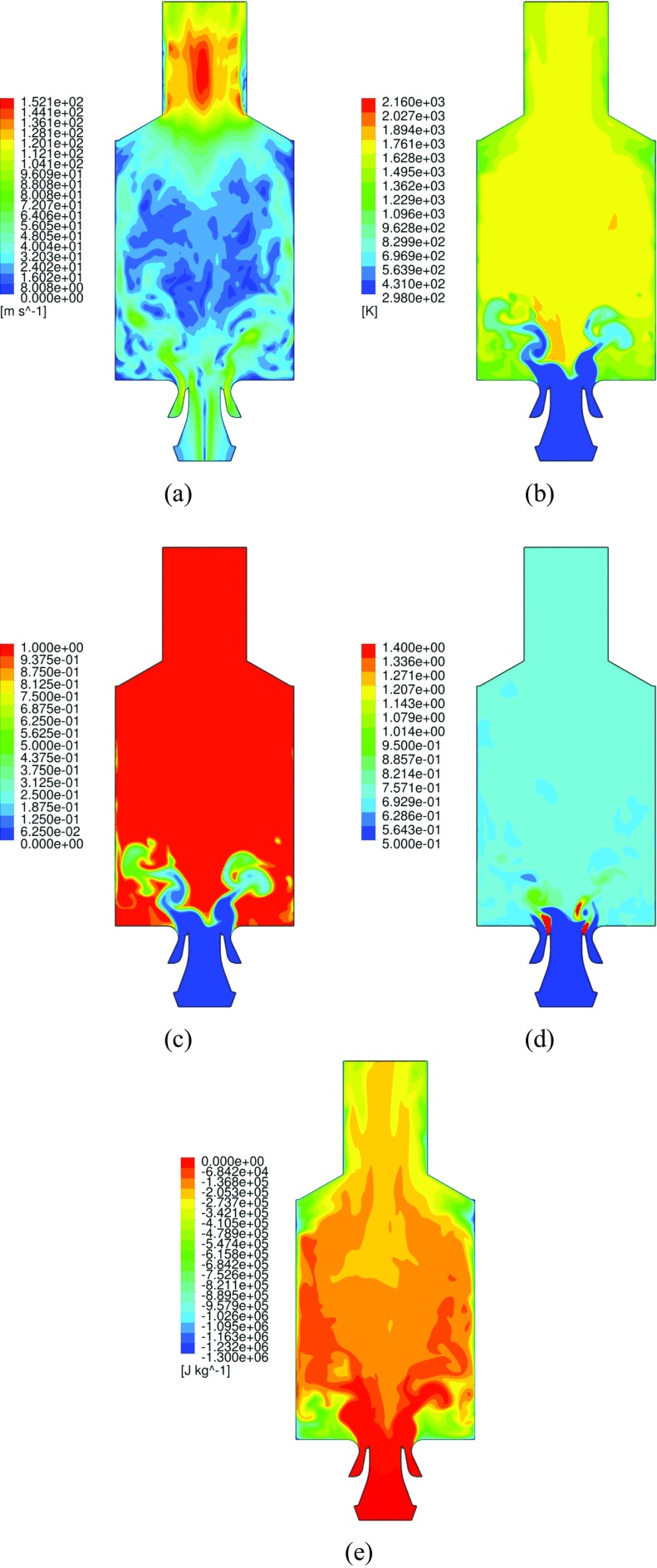
Iso-contour representations of the instantaneous flow-field distribution. Velocity magnitude (**a**) [m s ^−1^], temperature (**b**) [K], normalized progress variable (**c**), local equivalence ratio (**d**) and enthalpy loss (**e**) [J kg ^−1^] profiles at the midplane

**Fig. 7:**
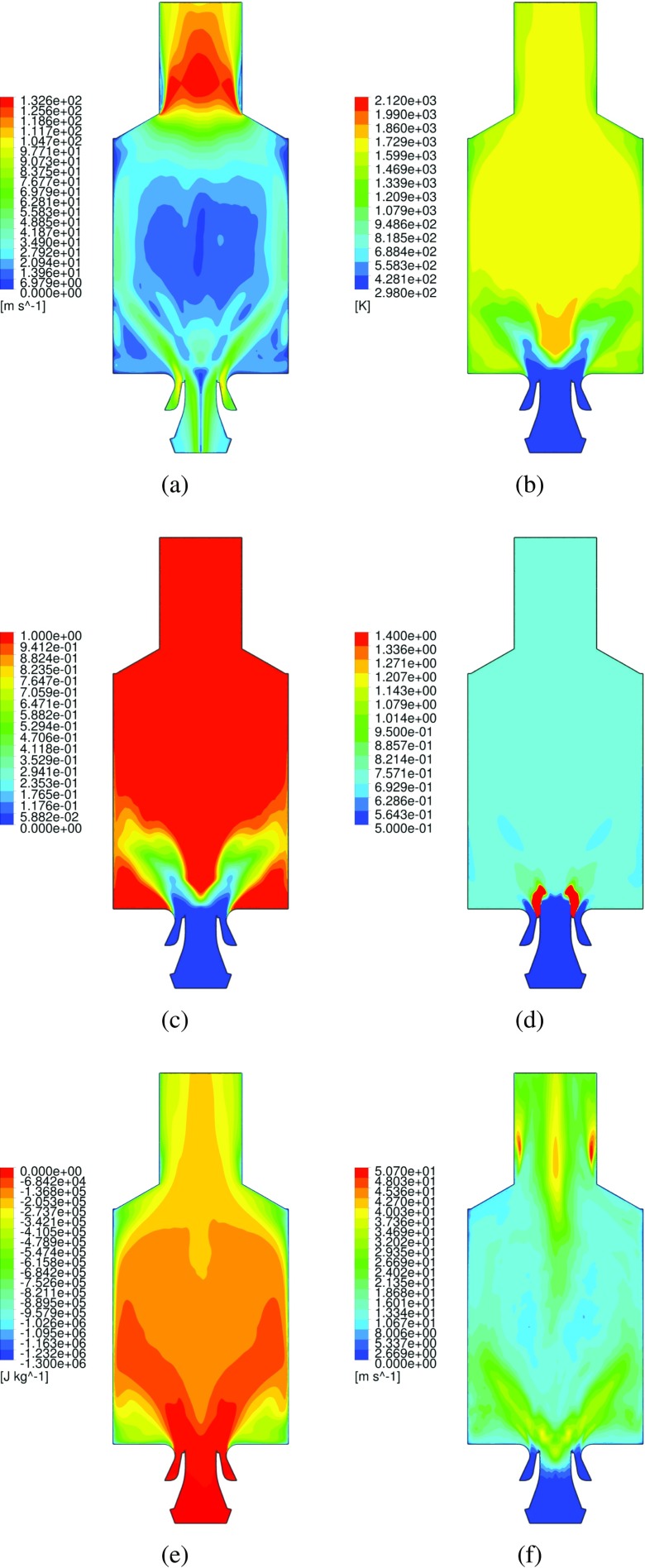
Iso-contour representations of the time-averaged flow-field distribution. Velocity magnitude (**a**) [m s ^−1^], temperature (**b**) [K], normalized progress variable (**c**), local equivalence ratio (**d**), enthalpy loss (**e**) [J kg ^−1^] and velocity standard deviation [m s ^−1^] profiles at the midplane

**Fig. 8:**
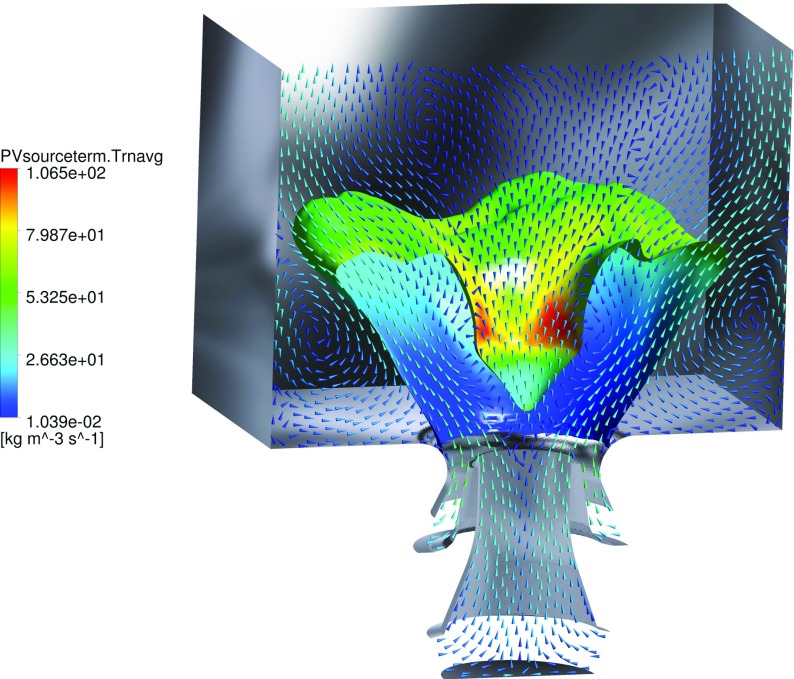
Representation of the time-averaged flow-field pattern. Iso-surface of $\mathcal {Y}$=0.6 colored by the progress variable source term. The vectors are representing velocity projection at the midplane, and colored as a function of the velocity magnitude

The global structure of the flame and the main flow field characteristics can be seen in the instantaneous and averaged flow profiles at the midplane, as given respectively in Fig. 6 and 7. The flame base appears highly corrugated and strongly affected by the instantaneous local flow conditions. As introduced in the representation of Fig. 3, the typical features of confined swirl-stabilized flames are observed. The stream of inlet gas forms a cone-shaped flow which extends to the outer wall, while along the axial center-line above the nozzle a strong inner recirculation zone (IRZ) opposes to the inlet gas stream, and an outer recirculation zone (ORZ) is observed externally to the nozzle near the walls at the dump plane edges, as depicted in Fig. 8. High velocities occur close to the nozzle exit and near the exhaust duct because of the significant area reduction. Very strong velocity gradients are present at the inner shear layer (between the IRZ and the inflow cone) and in a minor way at the outer shear layer (between the ORZ and the external side of the inflow cone). Important velocity fluctuations occur in these strong shear layers, as shown by the standard deviation given in Fig. 7f, but also in the zone that stretches along the centerline up to the outlet. These fluctuations are associated with two vortices: one vortex generated at the inlet and conically develops into the domain by rotating with a precessing mode, and an elongated vortex that forms in the exhaust duct contraction and extends back deep into the burner along the swirl axis location. A more detailed explanation of the characterization and motion of these vortices is given in Section 4.3.

### Combustion model assumptions verification

#### Flame mode

**Fig. 9:**
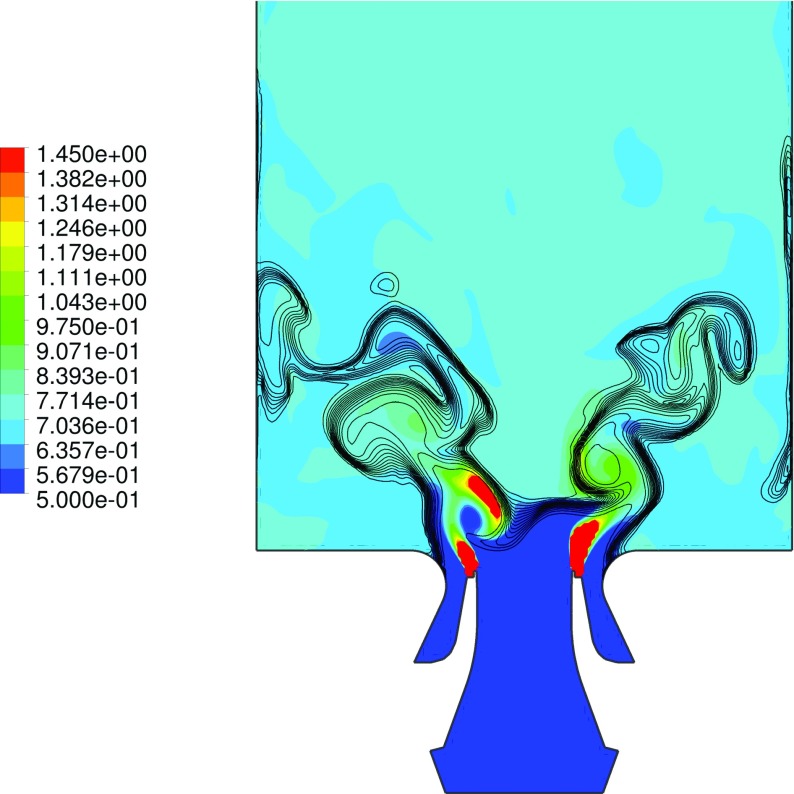
Representation of the instantaneous flow field at the midplane. *Colored contours* represent the local equivalence ratio, while the *black lines* are iso-contours of progress variable

A distinctive feature noticeable from e.g. Fig. 6c is that the flame does not result attached to the nozzle but rather oscillates at a lift-off height of several millimeters downstream. This is an indication of the high mixing level occurring before ignition, thus confirming the partially premixed nature of the reaction. This observation is furthermore supported by the experimental findings [28, 29]. In order to further assess this consideration, it is useful to examine the level of mixing at which the reaction is occurring. Figure 9 displays a snapshot of the local equivalence ratio at the midplane, together with iso-contours of progress variable in order to reveal the flame location (note that iso-progress variable lines does not alwats represent flame position since mixing between fresh and burnt gases can occurs without burning where the enthalpy level is low enough). The inlet geometry and conditions provide an extremely fast mixing, and therefore the reaction happens at equivalence ratios within the premixed flammability limits. Nonetheless, it is evident how pockets of not completely mixed fluid (i.e. which have higher equivalence ratio than the average *ϕ*_*global*_) are formed in a region located not far from the nozzle. These pockets are enclosed in large vortices, which are generated by the strong shear layer present at the interface between the IRZ and the inlet stream. The formation of these vortices (and therefore of the richer mixture pockets) happens at an almost steady vertical distance from the nozzle exit, but non-uniformly in the circumference as seen in Fig. 9. In fact this vortex is rotating with a certain frequency (see Section 4.3). However, the pockets of richer mixture are mixing very rapidly, and therefore richer combustion happens only in a limited region. In the averaged results this process leads to the formation of an annular band in which the equivalence ratio reaches values as high as 0.9. This consequently leads to higher local temperatures and reaction source terms, but also higher generation of NO, as discussed in Section 4.5. This phenomenon is evident in Fig. 8, in which it is visible how the averaged source term (the color of iso-surface of progress variable) is higher in this specific annular region.

**Fig. 10:**
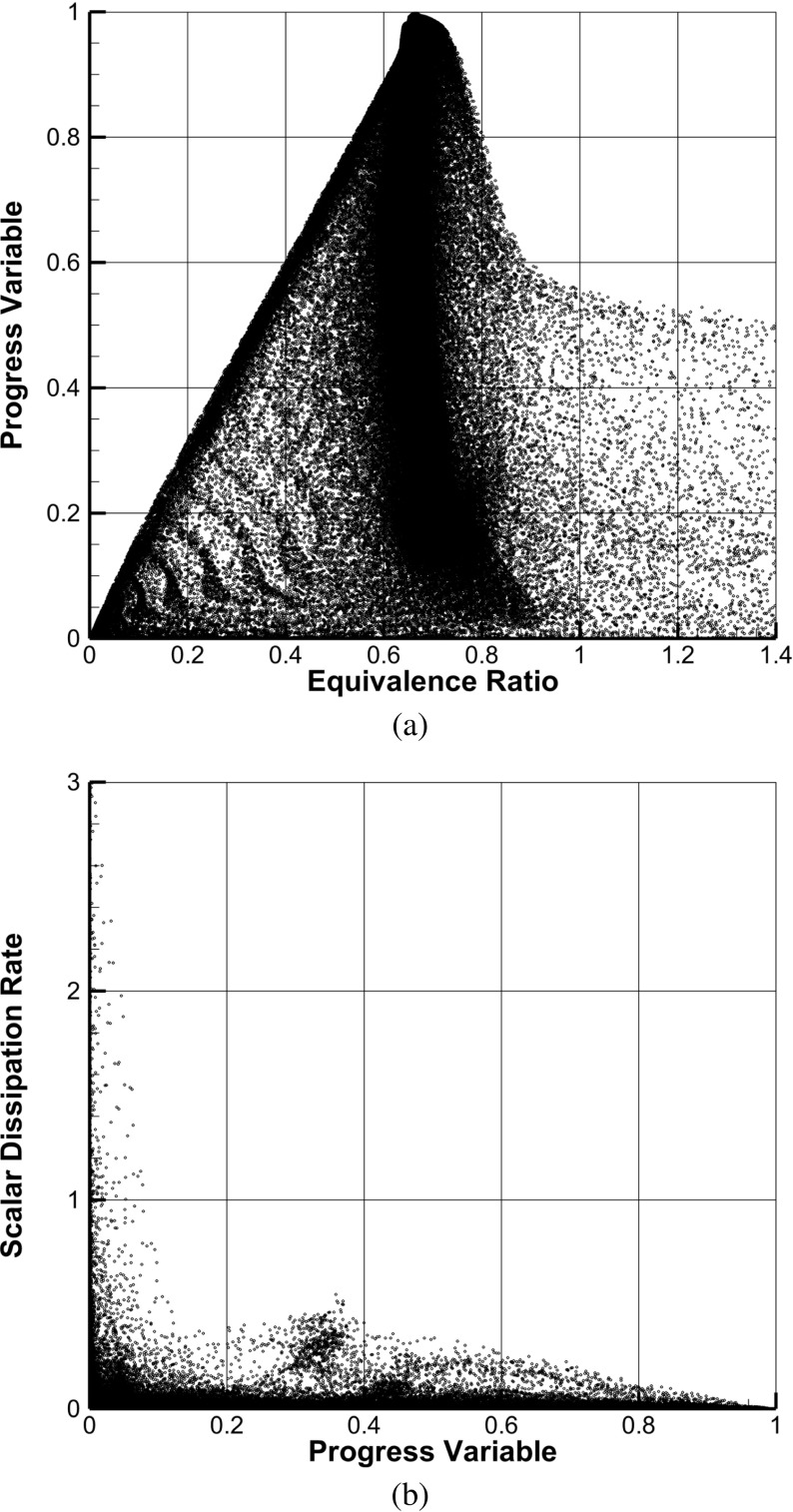
Scatter representations of the averaged flow field. **a** Normalized progress variable as a function of the local equivalence ratio. **b** Resolved scalar dissipation rate [1/s] versus progress variable

This consideration is further demonstrated by Fig. 10a, which shows a scatter plot of the averaged local equivalence ratio versus the averaged progress variable. This figure represents an important resource in order to assess at which level of mixing is the reaction happening. In the center of the figure it is noticeable a very high concentration of points in a semi-vertical band wide approximately 0.2 in terms of equivalence ratio. This band is located on top of the average value *ϕ*_*global*_, and it is representative of the reaction happening at the inner part of the domain. In this area of the scatter plot, the richer mixtures are found at lower values of progress variable. The curvature of this band reveals how a small part of the mixing still happens along the reaction. Furthermore, at progress variable $\mathcal {Y} =$ 1 the averaged equivalence ratio is recovered. At the left side of Fig. 10a another cluster of points is present along an inclined but almost rectilinear line. This area is representative of the mixing of fresh inlet air with the burned products happening at the ORZ. In general, in the ORZ the observed composition is relatively lean (*ϕ*<*ϕ*_*global*_). Only few active reaction points are located in the zone of higher equivalence ratios (*ϕ*≥1.4), namely less than 1 % which can be considered statistically not relevant. From this image the combustion type can be safely assumed as premixed.

Important information about the mixing level can also be captured by analyzing the rate of the fuel-air mixing in comparison with the progress of the reaction. In order to measure the rate at which the mixing is happening, the filtered scalar dissipation rate *χ* can be calculated by means of the first-order model as given in [93]:

13 $$ \widetilde{\chi} = 2\left( D_{Z} + D_{t} \right) \left( \nabla \widetilde{Z} \right)^{2},  $$

in which *D*_*Z*_ is the mixture fraction laminar diffusivity and *D*_*t*_ is the eddy diffusivity. The scalar dissipation rate is shown in Fig. 10b, in which the scatter plot of the averaged scalar dissipation rate as a function of the progress variable is given. This figure reveals a very important aspect about the mixing. It is directly noticeable that along the flame the scalar dissipation rate assumes very low values (for diffusion flames $\widetilde {\chi }$ typically assumes values of $\mathcal {O}(10^{2})$, e.g. [94]), meaning that the mixing is almost complete before the beginning of the reaction. In fact, the highest values (scalar dissipation rate of $\mathcal {O}(10^{2})$) are reached at $\mathcal {Y}=0$, and are out of this figure because of clarity of the representation. This corroborates the initial assumption regarding the premixed nature of the current simulations.

#### Flamelet hypothesis

A consideration about the scales involved in the flow and combustion process of the present case must be made in order to ensure that the flamelet hypothesis is valid [37]. The interaction between flow and chemistry may be evaluated through the analysis of the Karlovitz (*K* *a*≡*τ*_*c*_/*τ*_*k*_) and Damköhler (*D* *a*≡*τ*_*f*_/*τ*_*c*_) numbers (see e.g. [39] for an extended discussion on this topic). Here *τ*_*c*_, *τ*_*k*_ and *τ*_*f*_ respectively denote the time scales of chemistry, flow and smallest turbulent eddies (Kolmogorov). However, the calculation of these time scales is arbitrary to some extent, since both the flow field and chemical reactions cover a considerable range of scales. The chemical time scale *τ*_*c*_ is calculated as the ratio of the reaction zone thickness *δ*_*r*_ (calculated as proportional to the laminar flame thickness, as described in [53]) and the laminar flame speed *s*_*L*_, *τ*_*c*_ = *δ*_*r*_/*s*_*L*_. For a premixed methane/air free adiabatic flame at equivalence ratio *ϕ*=0.65 (average value in the domain) and atmospheric pressure, it results *δ*_*r*_=0.022 mm and *s*_*L*_=0.15 m/s. These values are calculated by means of a 1D detailed chemistry flat adiabatic flame simulation performed with [54]. In this way, it results in *τ*_*c*_≈0.15 ms. The flow time scale *τ*_*f*_ is chosen as the period of the vortex core precession, which essentially is not a turbulent process, (see Section 4.3 for further details on this flow feature), *τ*_*f*_=1/*f*_*PVC*_. The value of *f*_*PVC*_ was determined in the experiments of [69] as *f*_*PVC*_≈1500 Hz. The Kolmogorov time scale *τ*_*k*_ is estimated by expressing the dissipation rate in terms of large scale flow features. In this way, the large eddy turnover time can be assumed as the length of the large scale motion divided by its characteristic speed, and the eddy dissipation rate can be given as a function of the large scales [64, 95]. With this estimate, the Kolmogorov time scale can be calculated as $\tau _{k} = \tau _{f} / \sqrt {Re}$. Therefore, for the current case it results in *D* *a*≈4 and *K* *a*≈55. Here single values are not very representative and the given *K* *a* could be considered on the limit of the “broken reaction regime”. The validity of the full existence of flamelets, and its consequences, could be discussed.

**Fig. 11:**
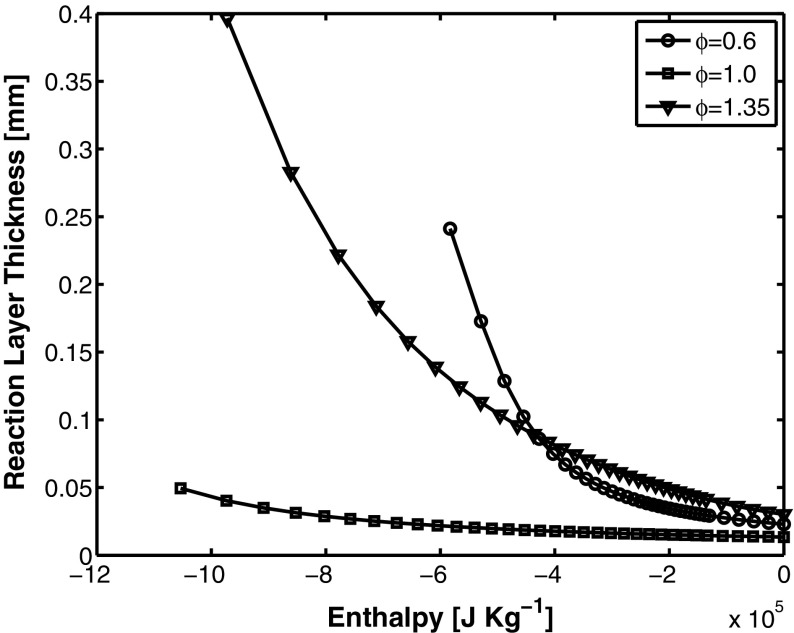
Flame reaction layer thickness as a function of the enthalpy of the flamelet. Each line corresponds to a different equivalence ratio

These values are subject to a number of uncertainties, given that the flame thickness is not constant throughout the domain. These variations in the flame thickness (i.e. and speed) are due to the non uniform fuel-air mixing and the heat loss. To this point, it is interesting to investigate the effect of mixture fraction and heat loss on the flame thickness. Figure 11 displays the reaction layer thicknesses *δ*_*r*_ as a function of the enthalpy of the laminar flamelet used for the FGM table construction. Every line corresponds to a different equivalence ratio (i.e. mixture fraction) and only three cases are shown in order to simplify the representation. The reaction layer becomes thicker with a decrease of enthalpy (i.e. with an increase of heat loss). This indicates a difference in the scales between the chemical reaction and the dissipative scales of the flow, with respect to adiabatic conditions. In the same way, the thermal thickness is minor at adiabatic conditions. This effect leads to more extended preheating zones in the cooled regions, e.g. next to the wall. The direct consequence of this behavior is that in all cooled regions the flow modeling is additionally crucial in order to have a correct flame location prediction. Regarding the mixture fraction effect on the flame, as expected the reaction thickness is minimum at stoichiometric conditions. In addition to this, heat loss seems to affect only in a minor way the flame thickness. Contrarily, for both lean and rich cases the flame thickness increases substantially with the decrease of enthalpy, and this effect is notably more marked in lean flames. The overall increase of the reaction thickness at the lowest enthalpy levels observed in the current simulations is of approximately 5–10 times, which would mean approaching the limit of the flamelet regime. Fortunately, in the present case the reaction is rarely happening in regions subject to high heat loss. In addition to this, in those locations turbulence is moderate, making us conclude that the flamelet hypothesis is respected in the simulations presented in this paper.

**Fig. 12:**
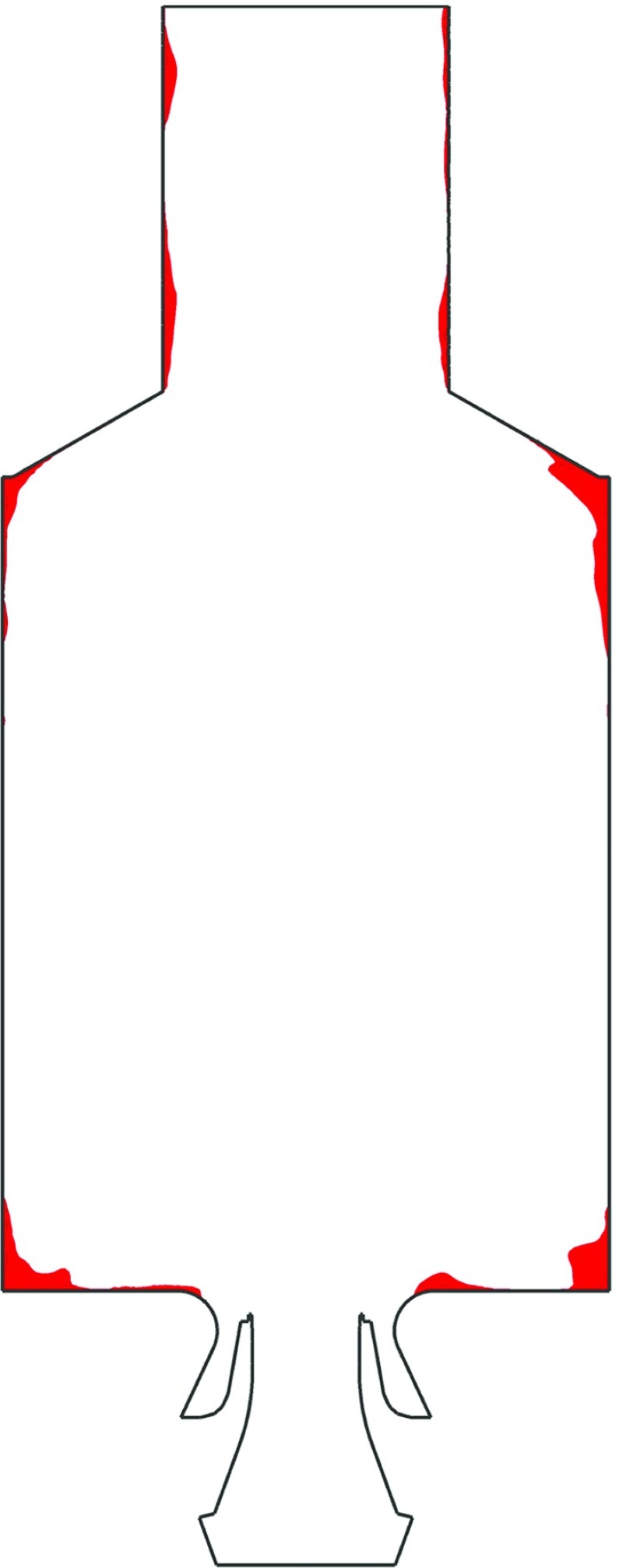
Instantaneous representation of the locations (in *red*), at the midplane of the domain, in which the sub-cooled region of the FGM database is accessed

As noted in Section 2.1, there is a region of the FGM database in which the enthalpy level lies between the limit of flamelet quenching due to heat loss and the minimum enthalpy. This area is defined as the sub-cooled region. Figure 12 displays in red the parts of the domain in which the enthalpy level lies in this particular region. Notably, the sub-cooled part of the FGM database is accessed only a in a very small part of the domain. This furthermore confirms that the basic modeling of this region is not a primary concern.

### Vortex core characterization and flame interaction

#### PVC precession frequency

**Fig. 13:**
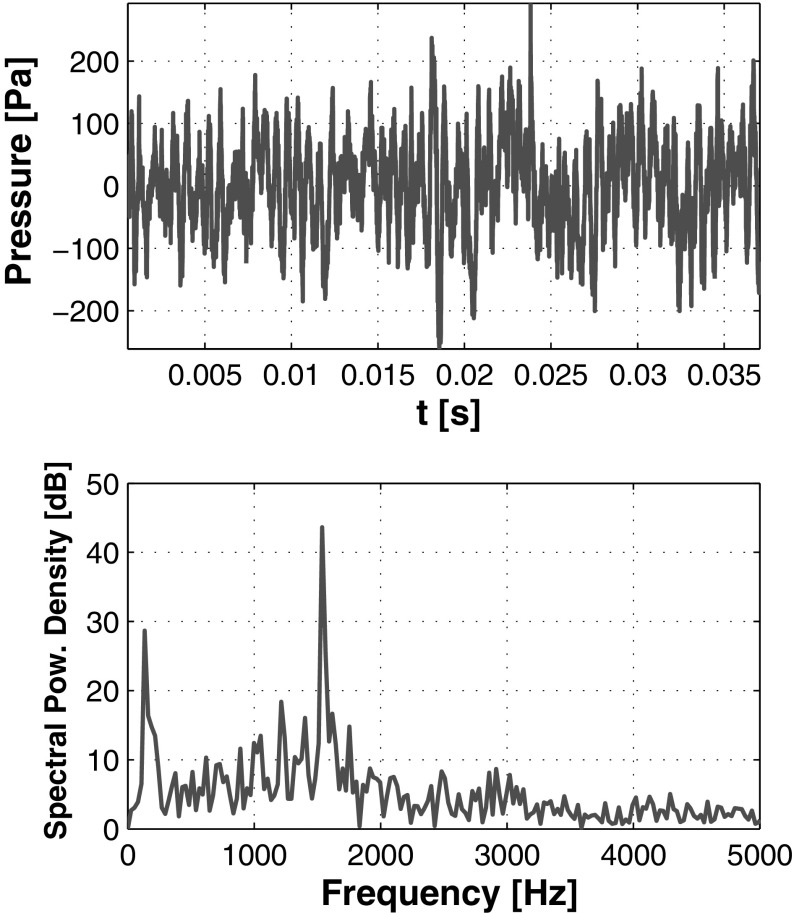
*Above*: pressure difference signal Δ*p*(*t*) between the two probes. *Below*: corresponding pressure spectrum

In [69] the PVC precession frequency of this burner was determined by using the pressure signals *p*_1_(*t*) and *p*_2_(*t*), which were measured with two probes at opposite corners of the combustion chamber at a height of $\mathcal {H} = 15$ mm from the dump plane. In order to maintain a consistent method of evaluation, the same is adopted in the present paper (albeit other methods are available in literature). Figure 13(top) shows the pressure signal of the difference Δ*p*(*t*) = *p*_1_(*t*)−*p*_2_(*t*) for the current LES simulation. The difference of the two signals is adopted here in order to cancel out the broad-band emissions and obtain a peak in the spectrum that can be clearly linked to the PVC. This is because the difference signal Δ*p*(*t*) is originated from an off-axis location that is pivoting around the center, while the broadband emissions may be mostly considered as axial-symmetric. Figure 13(below) shows the spectrum of the difference signal Δ*p*(*t*), and a single clear peak remains at *f*≈ 1500 Hz. This peak corresponds to the precession frequency of the PVC, and it results consistent with the experimental findings of [69]. This outcome is additionally confirmed by a manual count of the PVC rotations, which is obtained by the analysis of a series of instantaneous pressure fields at an horizontal section of the domain. It is noted that the frequency at which the PVC precedes is the same throughout the combustor [96], in contrast to the rotational frequency of the vortex core.

#### Vortex-flame interaction

**Fig. 14:**
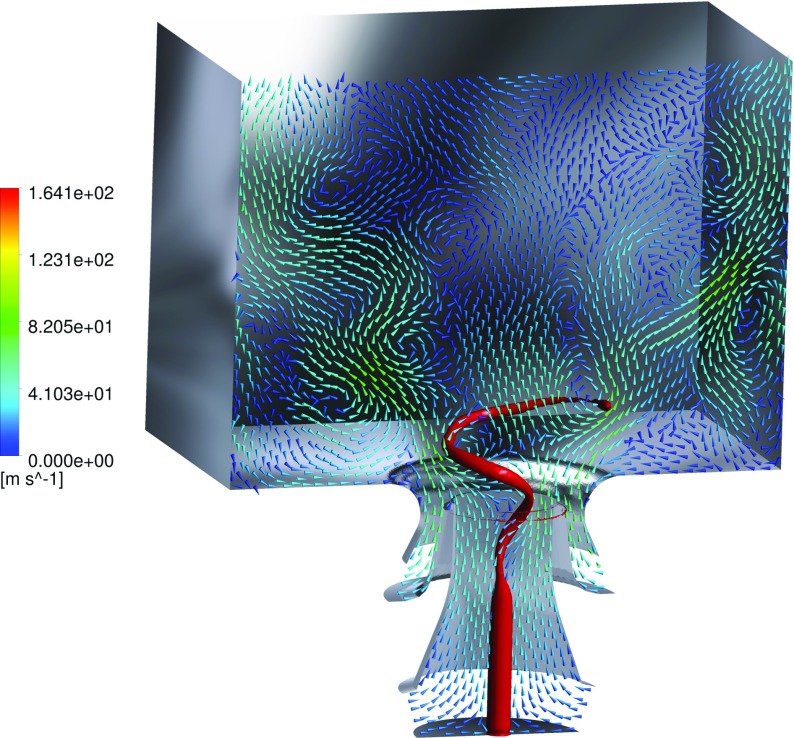
Representation of the instantaneous flow-field pattern. The *red iso-surface* displays the vortex core by means of the Q-criterion [97]. The vectors are representing velocity projection at the midplane, and colored as a function of the velocity magnitude

From the average flow-fields given in Fig. 7 it can be noted that the stagnation point of the IRZ (the bottom tip) is located in a very low position that reaches the dump plane level, whereas in the instantaneous flow fields given in Fig. 6 it appears to be located further downstream. The vortex originates in the axial region of the central air inlet duct, by means of the swirling motion of the inlet flow. The PVC initiates the rotation while approaching the inlet exit, enclosed in the rotating instability generated by the strong shear present at the interface of the inlet flow and the IRZ. This is shown in Fig. 14. The vortex rotates with a precessing mode, and thereafter conically expands until finally dissipating at the wall. It is important to notice that the averaged flow field of Fig. 7f indicates that the PVC leads to significant local variations of the swirling motion. In addition to this, the fact that the flame is stabilized at the inner shear layer suggests that the PVC strongly interacts with the flame. Another important feature is given by the exhaust tube vortex, highlighted by the strong velocity fluctuations along the center line of the upper chamber zone. These are generated by a vortex that forms in the exhaust pipe, and extends deep into the combustion chamber. A third helical vortex is found, located in the shear layer of the ORZ. However, in contrast with the PVC, the exhaust tube vortex and the ORZ vortex are relatively separated from the reaction zone, and therefore have no direct effect on the flame.

**Fig. 15:**
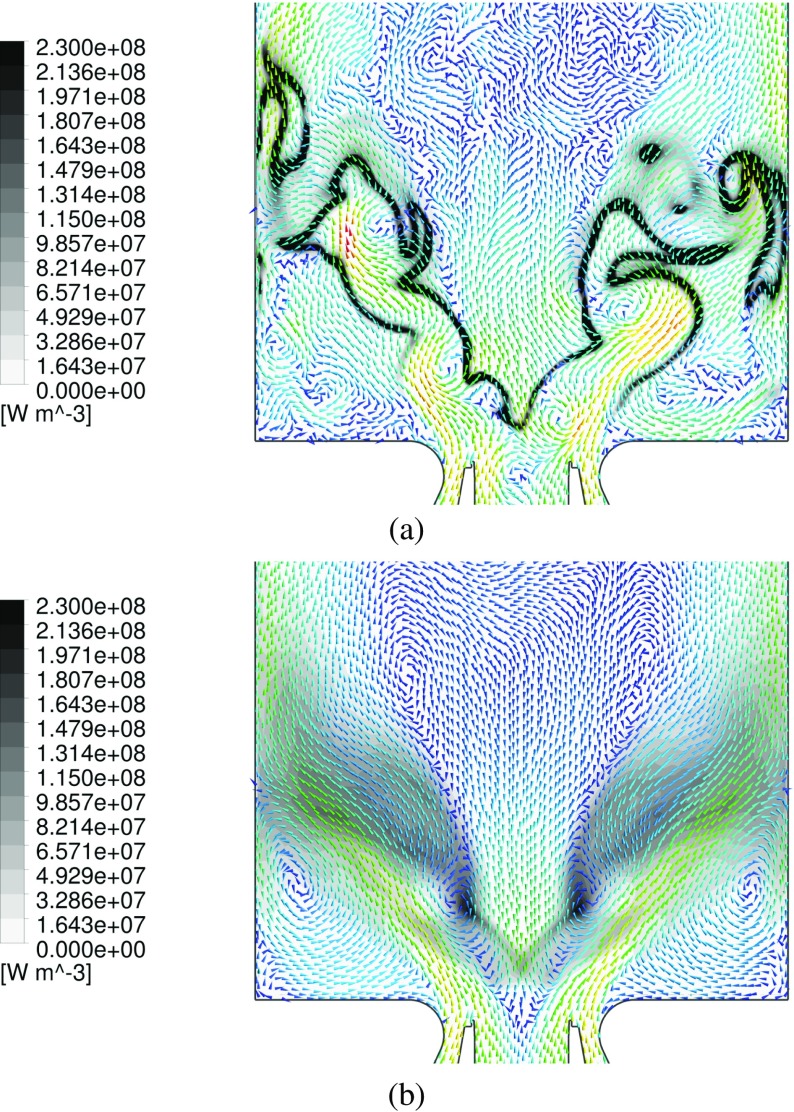
Iso-contour representation of the instantaneous (*top*) and averaged (*bottom*) heat release at the mid-plane. The same grey-scale range is adopted for both images. The vectors are representing velocity projection at the midplane, and colored as a function of the velocity magnitude

Figure 15 displays the combustion heat release profile from an instantaneous and averaged flow field. In addition, projected velocity vectors are superimposed in order to capture the vortex-flame interaction. In the IRZ it is observable how eddies initially cause deflections and partial folding of the reaction zone. This phenomenon greatly intensifies the supply of heat to the unburned mixture, hence favoring the impending ignition of the unburned gas. However, the folding (curving) of the reaction zone subsequently ends in a disruption of the flame. This folding-disrupting process is caused by the high velocities in proximity of the vortex center. This effect induces a local disruption of the flame, eventually leading to a collection of detached reaction zones further downstream. The present results demonstrate how strongly the PVC interacts with the flame and the importance of small scale features in the overall conversion process.

### Comparison with the experimental data

**Fig. 16:**
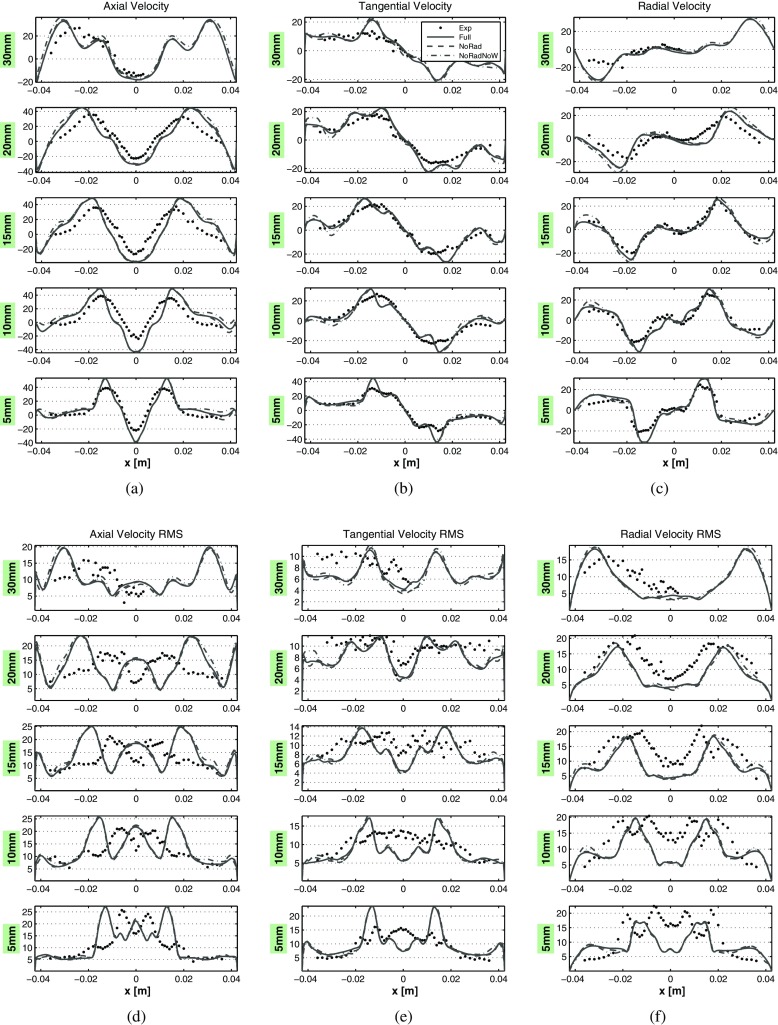
Averaged results comparison along central lines located at different axial distances from the nozzle. **a** Axial velocity [m s ^−1^]. **b** Tangential velocity [m s ^−1^]. **c** Radial velocity [m s ^−1^]. **d** Axial velocity RMS [m s ^−1^]. **e** Tangential velocity RMS [m s ^−1^]. **f** Radial velocity RMS [ m s ^−1^ ]. The *solid lines* represent the results obtained with the full model, the *dashed lines* are results obtained omitting radiative heat loss, the *dashed-dotted lines* represent results omitting both radiative and wall heat loss, while the *black dots* the averaged experimental data. The coordinate *x* here refers to the radial direction

A comparison between the profiles of transient averaged velocity and RMS components from the experimental data and LES is given in Fig. 16. The results are compared at five probe lines in the domain, which are located on the midplane at different distances from the reference plane, as described at the side of each plot. Error bars regarding the experimental data are not shown, given the modest error amount, as described in Section 3.1. In order to discriminate the effects of the heat loss on the results, three different LES are performed. One including the full model described in this paper, a second one with the same model but without the inclusion of radiative heat loss effects (therefore still including the effect of heat loss to the walls) and a third one with the same model but excluding both heat loss effects (i.e. adiabatic case). In each plot of Fig. 16 the results obtained with the full LES model are compared with the ones obtained omitting radiative heat loss and the ones omitting both radiative and wall heat loss, and finally the averaged experimental data. These are marked in the legend as “Full”, “NoRad”, “NoRadNoW” and “Exp” respectively.

As commonly observed in highly swirled flows, the sudden radial expansion of the velocity field right after the nozzle exit generates a pressure gradient. This pressure gradient causes the formation of the IRZ, which serves as flame stabilization mechanism. Regarding the axial velocity, the positive peaks are representing the inlet flow of fresh gases, while the negative peak is caused by the strong reversed flow induced by the IRZ. At the first measurement line ($\mathcal {H} = 5$ mm) the mean part is very well predicted, except for the peaks of axial and radial velocity which are overestimated. On the same location the fluctuating part is reasonably predicted, in fact the magnitude is very close (for all the components) to the experiments whereas the axial velocity RMS shape is not fully replicating the experimental one. This indicates that the artificial inlet forcing is capable to describe the turbulent structures without including the upstream geometry, but possibly it should be set to a different intensity for the two air inlets. Going to increasing distances from the nozzle the mean experimental results are still very well reproduced by the LES under study. The discrepancy from the experiments actually decreases at higher distances. Furthermore, the strong peaks of the RMS velocity indicate that the flow field undergoes strong turbulent fluctuations and that the shear layer is locally unstable. The main noticeable differences are an overall higher spreading of the turbulent swirling flow, and a higher IRZ backward velocity at the axial location. The swirling flow cone angle can be calculated on the base of the axial velocity peaks at the nozzle distances of $\mathcal {H} = 10-20$ mm, with respect to the axial line. In this way it results in ∼27^∘^ for the experiments and ∼34^∘^ for the LES results. The increased cone angle is the main source of error for the mean velocity results. Furthermore, it is reasonable to assume that the higher spreading of the turbulent swirling flow is the principal cause of the higher velocities in the IRZ. The inaccuracy of the cone angle prediction might be due to the inlet air split ratio (between air mass flowing through the annular and central nozzle) chosen for the present numerical study, which could be slightly different than the experimental one. However, this is pure speculation. Overall, the present formulation reproduces satisfactorily the mean flow-field.

The inclusion of heat loss does not affect the velocity profiles, especially in the region close to the dump plane. However, a difference is noticeable for higher distances from the nozzle, in which the heat loss seems to reduce the peaks. This is due to the lower temperatures, which lead to a lower density decrease due to the reaction and therefore reduced velocities. Notably, the velocity fluctuations at the IRZ are higher in the case of heat loss inclusion.

**Fig. 17:**
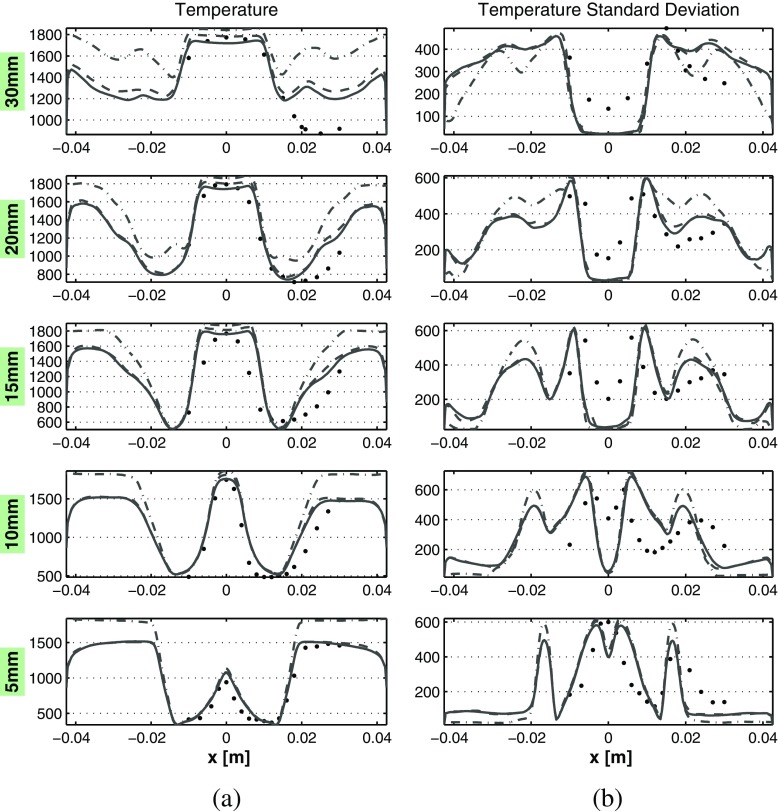
Averaged results comparison along central lines located at different axial distances from the nozzle. **a** Temperature [K]. **b** Temperature standard deviation [K]. The *solid lines* represent the results obtained with the full model, the *dashed lines* are results obtained omitting radiative heat loss, the *dashed-dotted lines* represent results omitting both radiative and wall heat loss, while the *black dots* the averaged experimental data. The coordinate *x* here refers to the radial direction

The transient average and RMS of temperature comparison between the profiles of the experiments and LES is given in Fig. 17. As delineated in Section 2.3, the temperature is purely derived from the tabulated data, therefore by means of a post-processing step. However, temperature results are important in order to indicate whether the controlling variables employed in the current FGM implementation are able to describe the physical situation. The averaged temperature profiles show how the flame stabilizes in the inner shear layer. The averaged temperature profiles are in general good agreement with the experiments. It is clearly noticeable how in the IRZ the fast mixing leads to averaged temperatures which are consistent with the burned value of laminar premixed flames at *ϕ* = *ϕ*_*global*_. However, the temperature standard deviation shows high fluctuations of the temperature especially in the inner shear layer, due to pockets of relatively richer mixture (*ϕ*>*ϕ*_*global*_). Here the temperature fluctuations are as high as 550-600 K close to the nozzle. In the contrary, at the ORZ the relatively lean mixture (*ϕ*<*ϕ*_*global*_) gives lower burned gas temperatures. This is further enhanced by the strong heat loss to the wall present at the ORZ. This is consistent to what is shown in Section 4.2.1. In general, the previously discussed inaccurate cone angle prediction is the main source of error for the temperature results. This leads to a smaller vertical extension of the ORZ, in fact causing the incorrect higher temperature prediction in this zone for $\mathcal {H} = 30$ mm. In addition, the wider spread of the flow leads to lower temperature deviations predictions in the axial region.

Heat loss inclusion has a strong impact on the temperature profiles, as expected. This effect is enhanced in the recirculation zones, but in different ways depending on the location. In fact, at the IRZ the radiative heat loss are more effective than the wall losses, given the high distance from the walls and the confinement among the inlet flow created by the swirling motion. Contrarily, wall heat loss are very strong at the ORZ, where differences as big as 300 K are observed between adiabatic and full model. In general, heat loss inclusion is necessary in order to correctly represent the experimental temperature profile. Notably, heat loss inclusion affects also the temperature fluctuations, especially at the ORZ and at higher distances from the nozzle exit.

The overall calculation time of LES is an important factor. The CPU time for the gas turbine combustor simulations shown in this paper is about 40000 CPU hours, which means approximately 320 wall clock hours in the machines adopted for the present case. The adopted machine was equipped with 16 and 32GB of RAM memory, according to the node. This amount of memory results to be sufficient for storing the 5D tabulated data during runtime. With the development of larger computing resources, it is foreseeable that LES may soon become the preferred tool for gas turbine applications.

### Emissions analysis

**Fig. 18:**
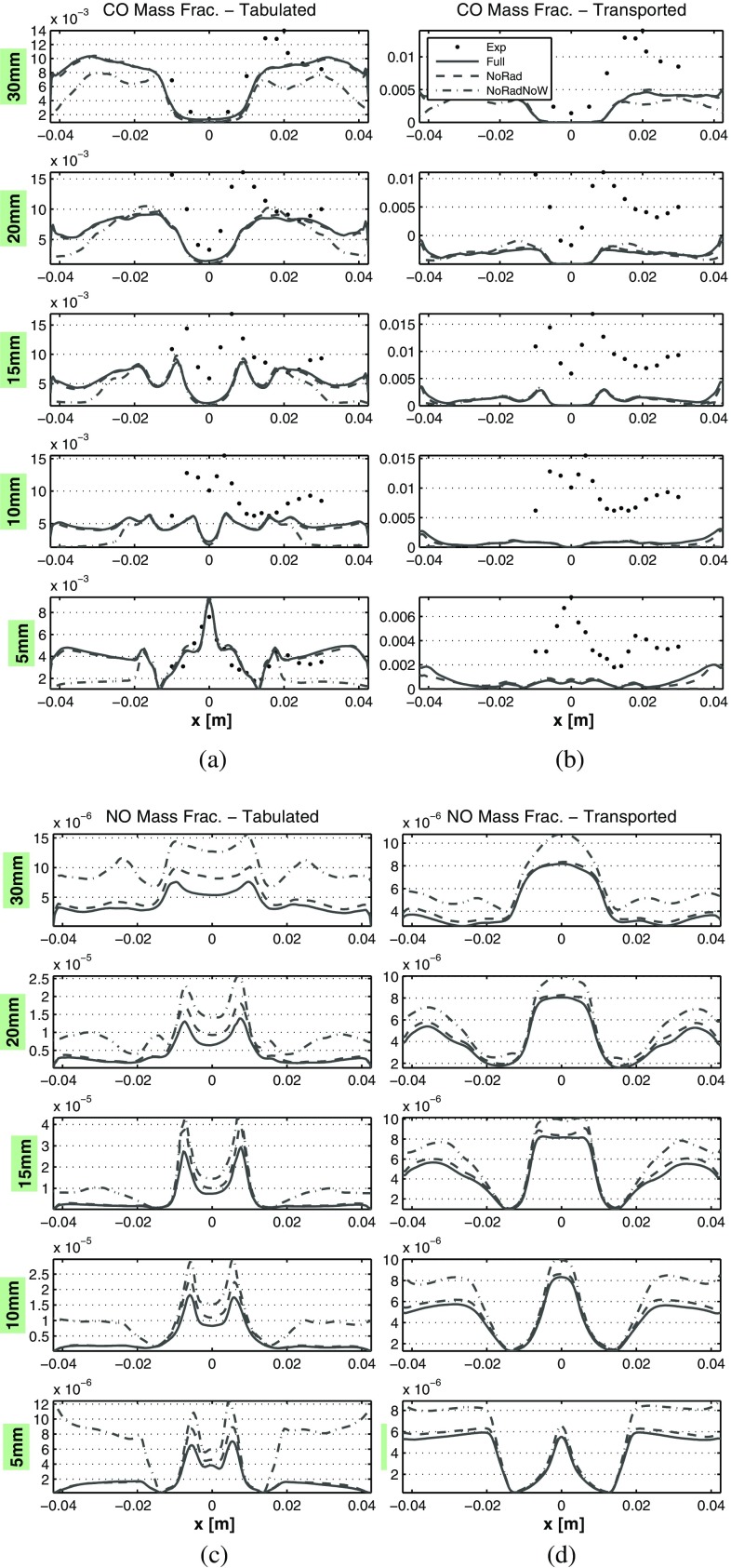
Averaged mass fractions comparison along central lines located at different axial distances from the nozzle. **a** CO from FGM. **b** CO transported. **c** NO from FGM. **d** NO transported. The lines description is described in the legend, and it is the same of Fig. 16

A comparison between the profiles of transient averaged CO and NO mass fractions is given in Fig. 18. Carbon monoxide profiles are compared with the experimental data, while nitric oxide results are compared only among simulations given that no experimental data is available for this species. Here two techniques for calculating slow pollutants with FGM are compared. On the left plots of Fig. 18 the mass fractions are directly retrieved from the tabulated data, while on the right plots these are calculated by means of two extra transport equations, as explained in Section 2.3. The averaged CO profiles of Fig. 18a and b are reasonably close to the experimental values for the tabulated method, even though there is a relevant difference at the high velocity locations at the swirl cone, especially at intermediate distances from the nozzle. In addition, the tabulated direct retrieval of CO shows to predict well the trend. Overall, the transported method for CO is not as effective as the direct retrieval from the FGM table. This is consistent with the results of [50], in which this approach has proven to yield improved predictions only for nitric oxide mass fractions.

In addition, the CO profiles for the transported methods are only slightly affected by the heat loss inclusion, whereas for the tabulated method this effect is stronger. In any case, for the tabulated method the simulation with full heat loss inclusion gives the closest CO match with the experimental data.

Figure 18c show the time averaged NO mass fraction profiles. In general, a large discrepancy is observed between the tabulated and transported results, not only in the overall quantities but also in terms of profiles conformation. This is because the generation of NO is governed by slow chemical processes and therefore cannot be properly described only by the current control variables. In fact, the NO mass fraction calculated by means of the extra transport equation has comparatively much higher values in the recirculation zones and especially in the IRZ. This is because the source term of NO is non zero in the burned gases (the formation of NO is very sensitive to temperature), especially where the temperatures are higher such as at the IRZ, and this effect can be reproduced only by means of the transported model. It has to be noted that the GRI-Mech 3.0 mechanism is used in this study, which is able to accurately describe both thermal NO (Zeldovich mechanism) and prompt NO (Fenimore mechanism) formation processes for methane/air flames [55].

Regarding the heat loss inclusion effect on NO prediction, again a big difference in behavior is observed between the tabulated and transported results. The tabulated method shows a bigger difference in the flame region with respect to the transported, as a result of the accumulated NO created at the recirculation zones. This history effect is not present in the tabulated method, and as a result of this the heat loss inclusion affects the differences in NO mass fraction in totally different zones.

**Fig. 19:**
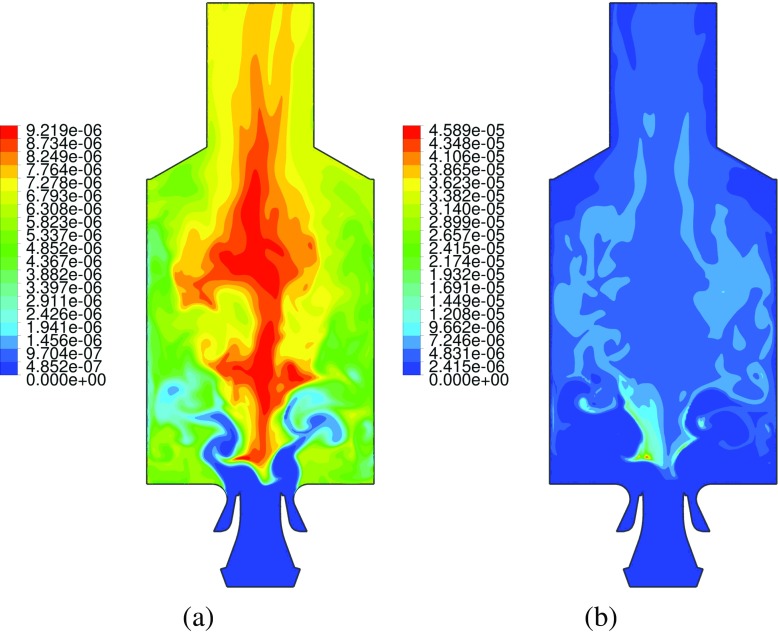
Iso-contour representations of the instantaneous NO mass fractions distributions at the midplane. **a** Transported NO. **b** Tabulated NO

Unfortunately the experimental data is lacking of NO measurements at inner locations of the combustion chamber. However, the nitric oxide composition measurement is available at the exhaust. This measurement resulted in a time averaged NO concentration of 6 parts per million (ppm). Table 1 gives the NO exhaust concentrations (averaged over time and over the exhaust area) for the solutions of the current simulation set. The amount of NO predicted by the full LES model with transported NO is in excellent agreement with the experimental data. The very small difference (less than 5 %) between this and the experiments is given by the slightly wrong prediction in the swirl cone angle. In fact a higher cone angle leads to an enlarged central recirculation zone (IRZ), and therefore a higher residence time for gas located in this high temperature zone (causing higher thermal NO). On the other hand, the NO exhaust predictions given by direct retrieval from the FGM table are three times lower than the experiments. This leads to conclude that a transport equation for NO is essential in order to correctly capture this pollutant. The difference of NO predictions obtained by these two methods are clearly visible also from the instantaneous fields given in Fig. 19. This figure reveals that the results obtained by the two methods are contrasting both quantitatively and in terms of location in the flame.

**Table 1:** Averaged nitric oxide emission at the exhaust [ppm] for the different simulations

	Full	NoRad	NoRadNoW
NO Tabulated	2.15	2.95	14.10
NO Transported	6.26	6.99	8.97

Experimental measurements reported a value of 6 ppm

Overall, the impact of heat loss on thermal NO is evident. The NO results obtained without radiative heat loss inclusion in the model are still reasonably good. This because the heat loss given by radiation is not so strong compared to the wall heat loss. In fact the NO predictions for the adiabatic model are far off the experiments. Furthermore, oppositely to what is observed for CO, it is clear how the direct tabulated retrieval method for NO is not as effective as the transported. Therefore, the slightly higher computational effort for the additional transport equation is justified by the high prediction accuracy for NO.

## Conclusions

The well established FGM combustion model is progressively extended for the inclusion of all the combustion features that are typically observed in stationary gas-turbine combustion. These consist of stratification effects, heat loss and turbulence. A highly turbulent and swirling flame in a gas turbine combustion chamber is computed by means of the present FGM implementation coupled to an LES turbulence model, and the results are compared with experimental data. In general, the model has demonstrated a rather good agreement with experimental data. It is shown that the inclusion of heat loss greatly improves the temperature predictions in the whole burner and leads to slightly better velocity predictions at downstream positions. As a result of the improved temperature predictions, the amount of NO predicted by the model is in excellent agreement with the experimental data. In addition, the inclusion of radiative heat loss in the model does not lead to a significant impact on the overall flame structure, but considerably affects NO predictions. This model sets the basis for the simulation and development of current and future gas turbine burners at high power conditions, for the alternative fuel usage in a cleaner and more efficient combustion.

## Footnotes

1
^1^ This categorization is not entirely inclusive, nonetheless still useful and representative for the largest part of the models available in literature.
